# Supplementary figures and images for: The color phi phenomenon: Not so special, after all?
Source: PLoS Comput Biol. 2021 Sep 3;17(9):e1009344. doi: 10.1371/journal.pcbi.1009344 (PMC8445478; doi:10.1371/journal.pcbi.1009344)

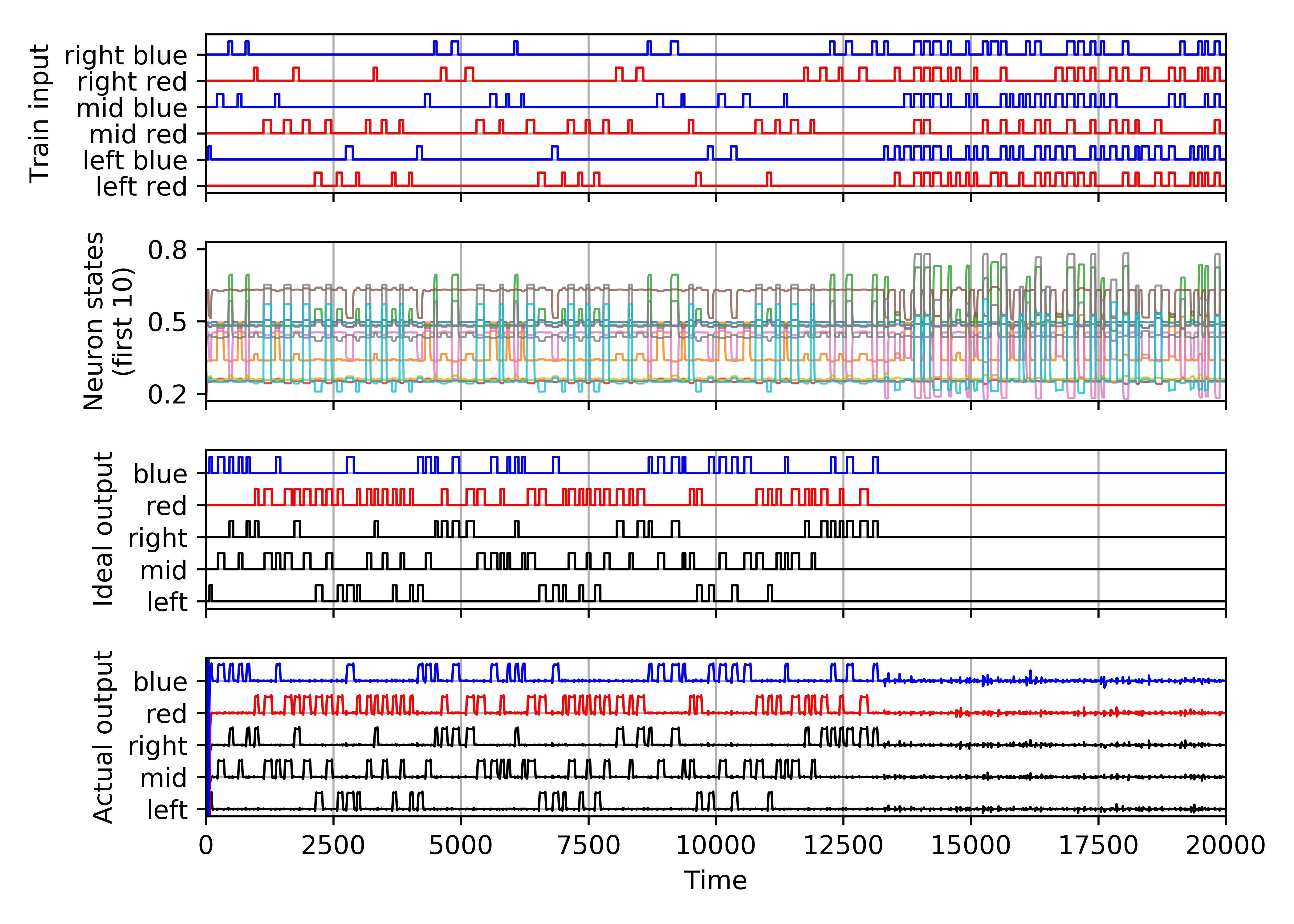

Supplement: S1 Data — (ZIP) [file pcbi.1009344.s005.zip › S1_Data/colphi_hires/Fig6.png]

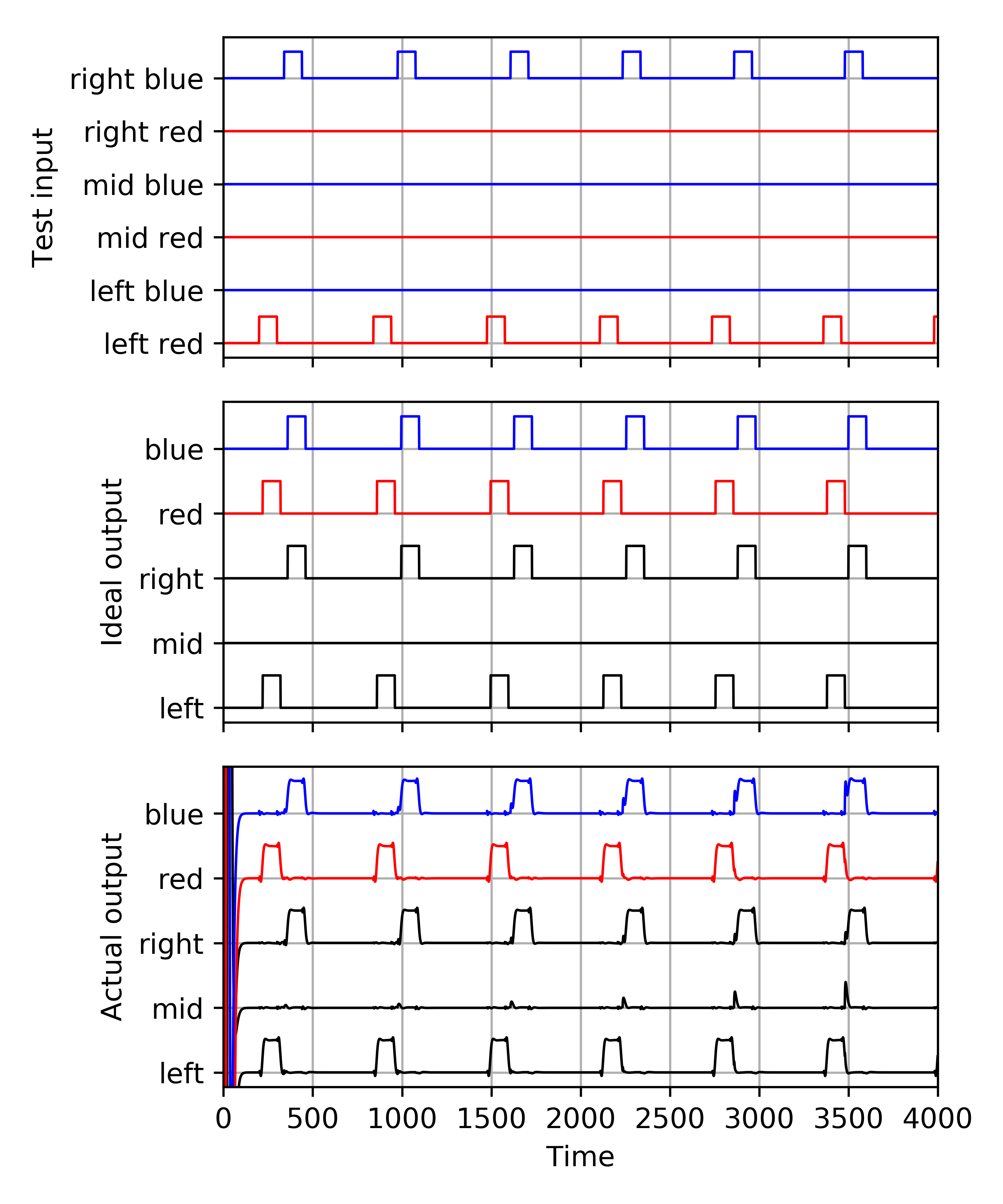

Supplement: S1 Data — (ZIP) [file pcbi.1009344.s005.zip › S1_Data/colphi_hires/Fig7a.png]

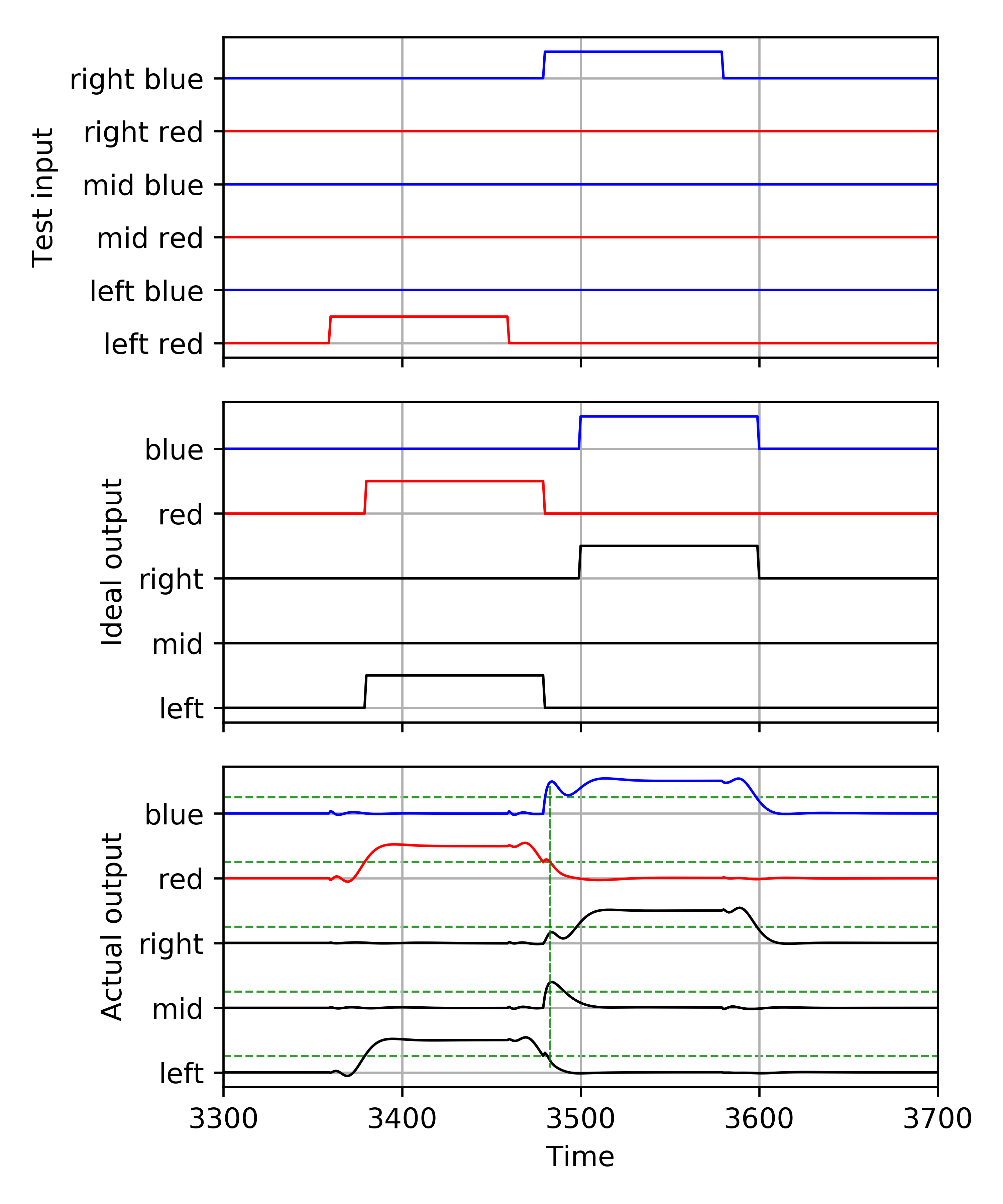

Supplement: S1 Data — (ZIP) [file pcbi.1009344.s005.zip › S1_Data/colphi_hires/Fig7b.png]

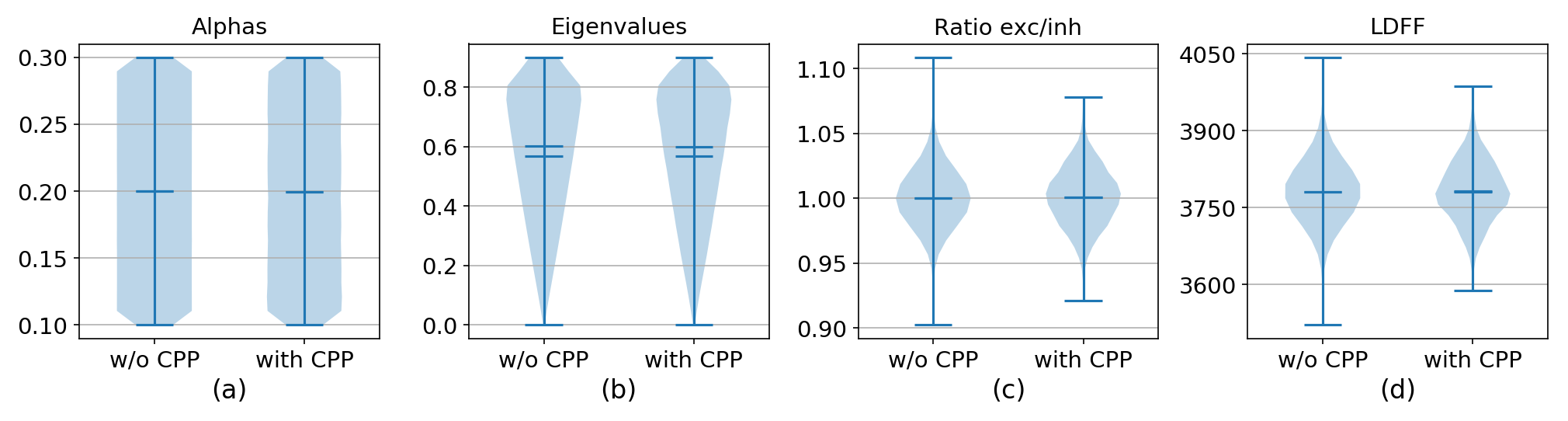

Supplement: S1 Data — (ZIP) [file pcbi.1009344.s005.zip › S1_Data/colphi_hires/extra_analysis/Fig8.png]

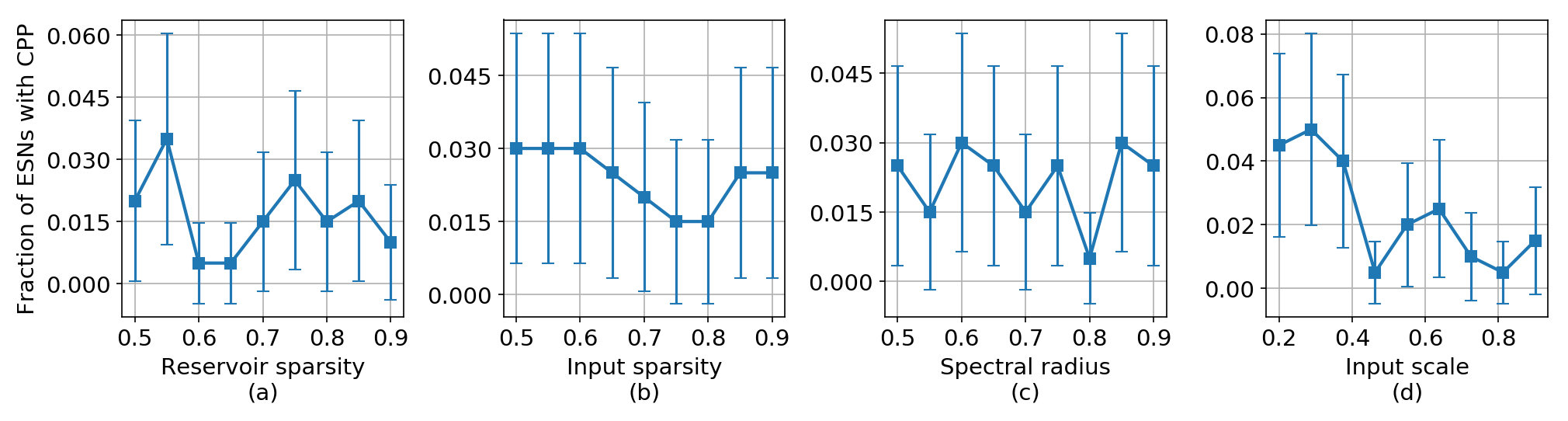

Supplement: S1 Data — (ZIP) [file pcbi.1009344.s005.zip › S1_Data/colphi_hires/extra_scans/Fig9.png]

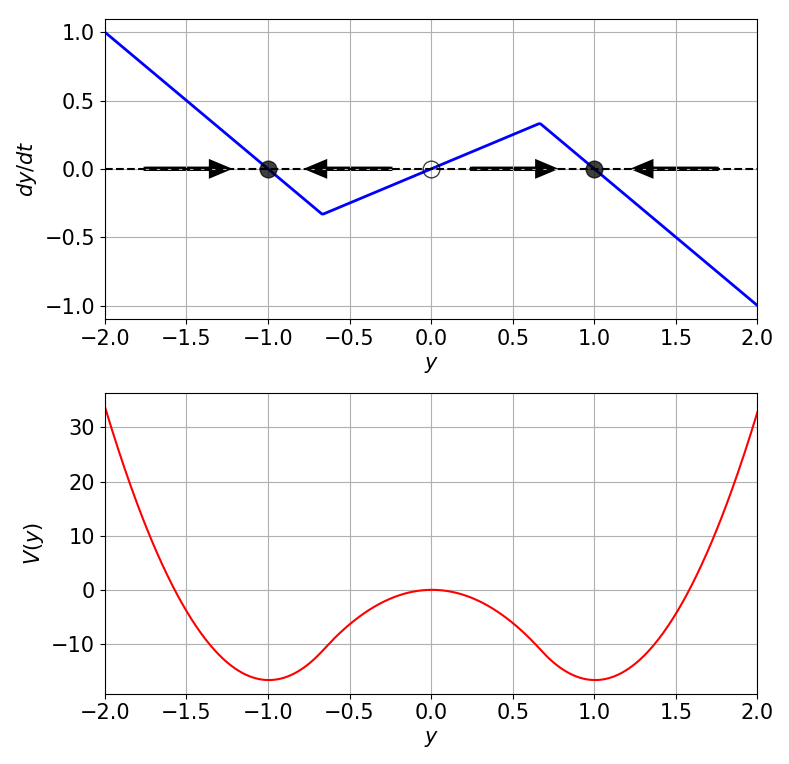

Supplement: S1 Data — (ZIP) [file pcbi.1009344.s005.zip › S1_Data/masking2/Fig2b.png]

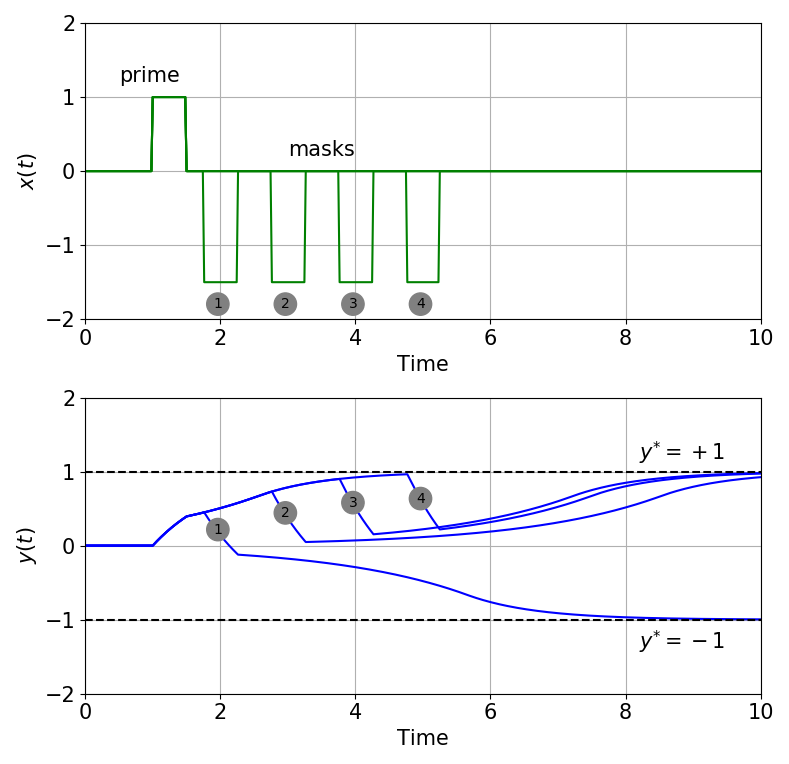

Supplement: S1 Data — (ZIP) [file pcbi.1009344.s005.zip › S1_Data/masking3/Fig2c.png]

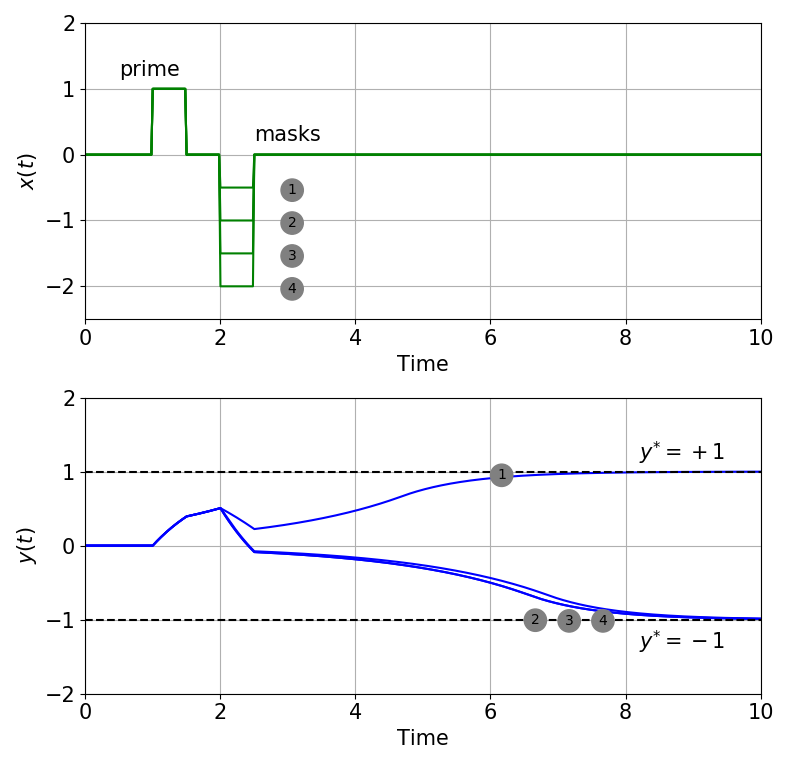

Supplement: S1 Data — (ZIP) [file pcbi.1009344.s005.zip › S1_Data/masking4/Fig2d.png]

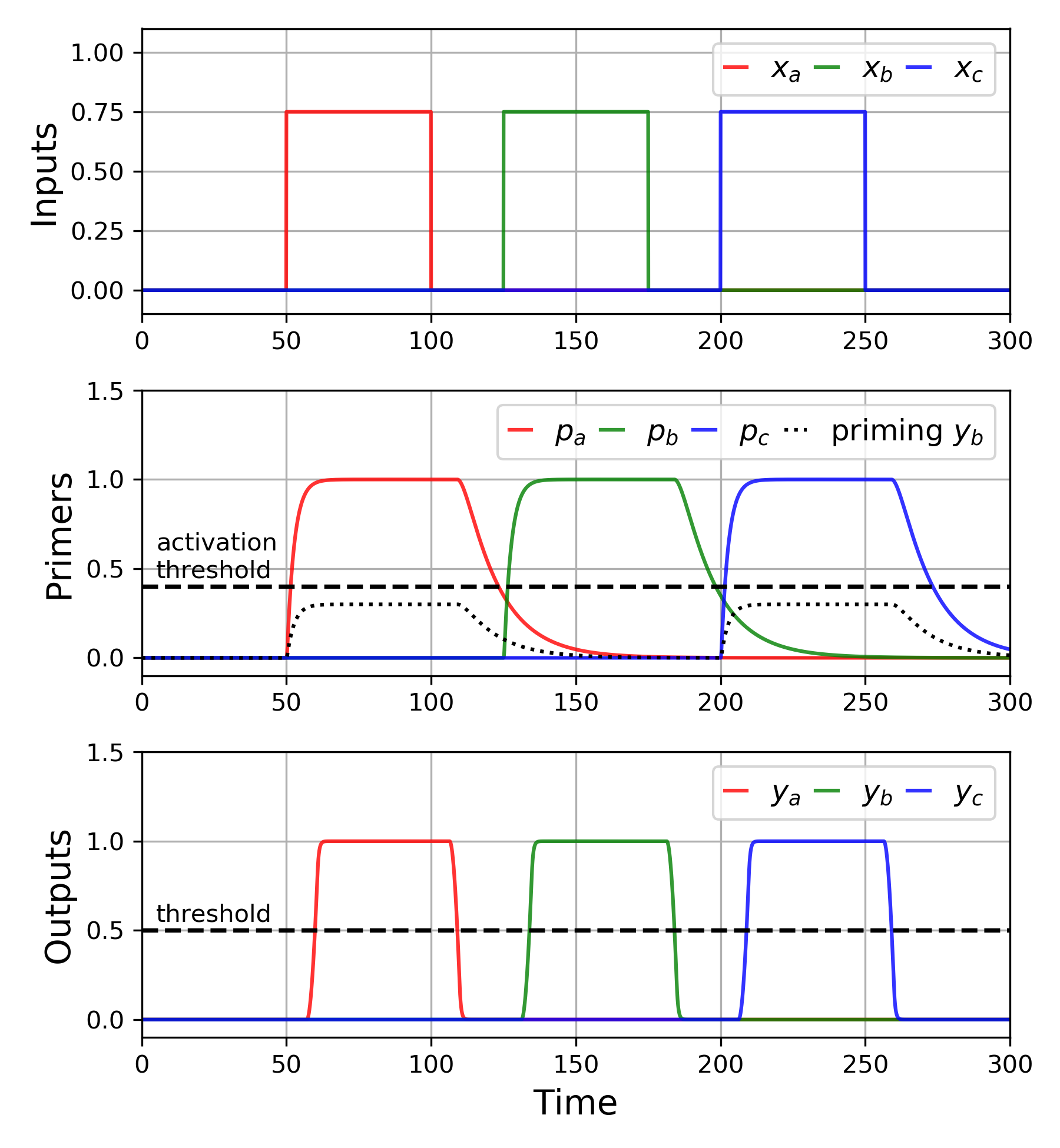

Supplement: S1 Data — (ZIP) [file pcbi.1009344.s005.zip › S1_Data/phi/Fig4b.png]

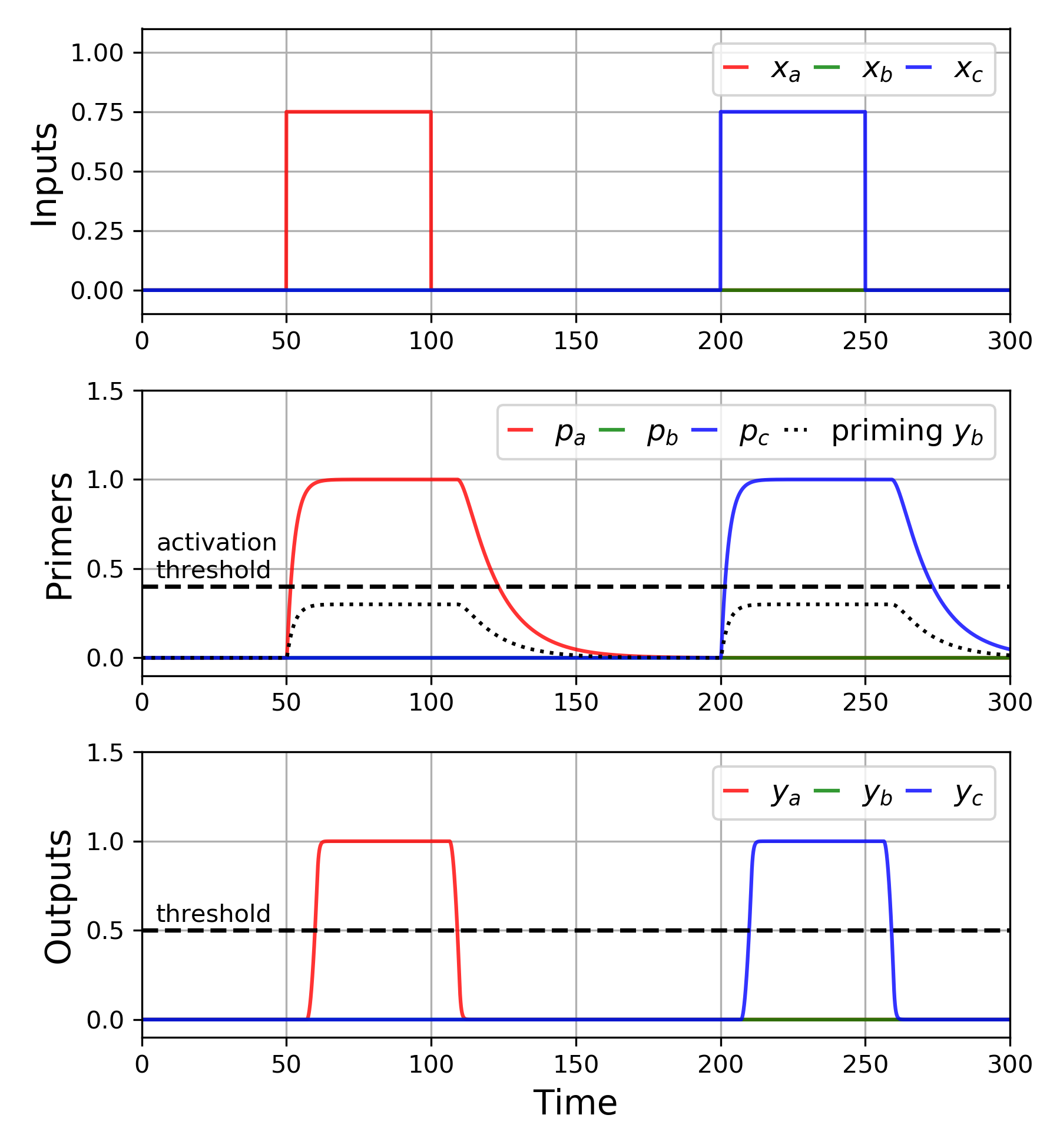

Supplement: S1 Data — (ZIP) [file pcbi.1009344.s005.zip › S1_Data/phi/Fig4c.png]

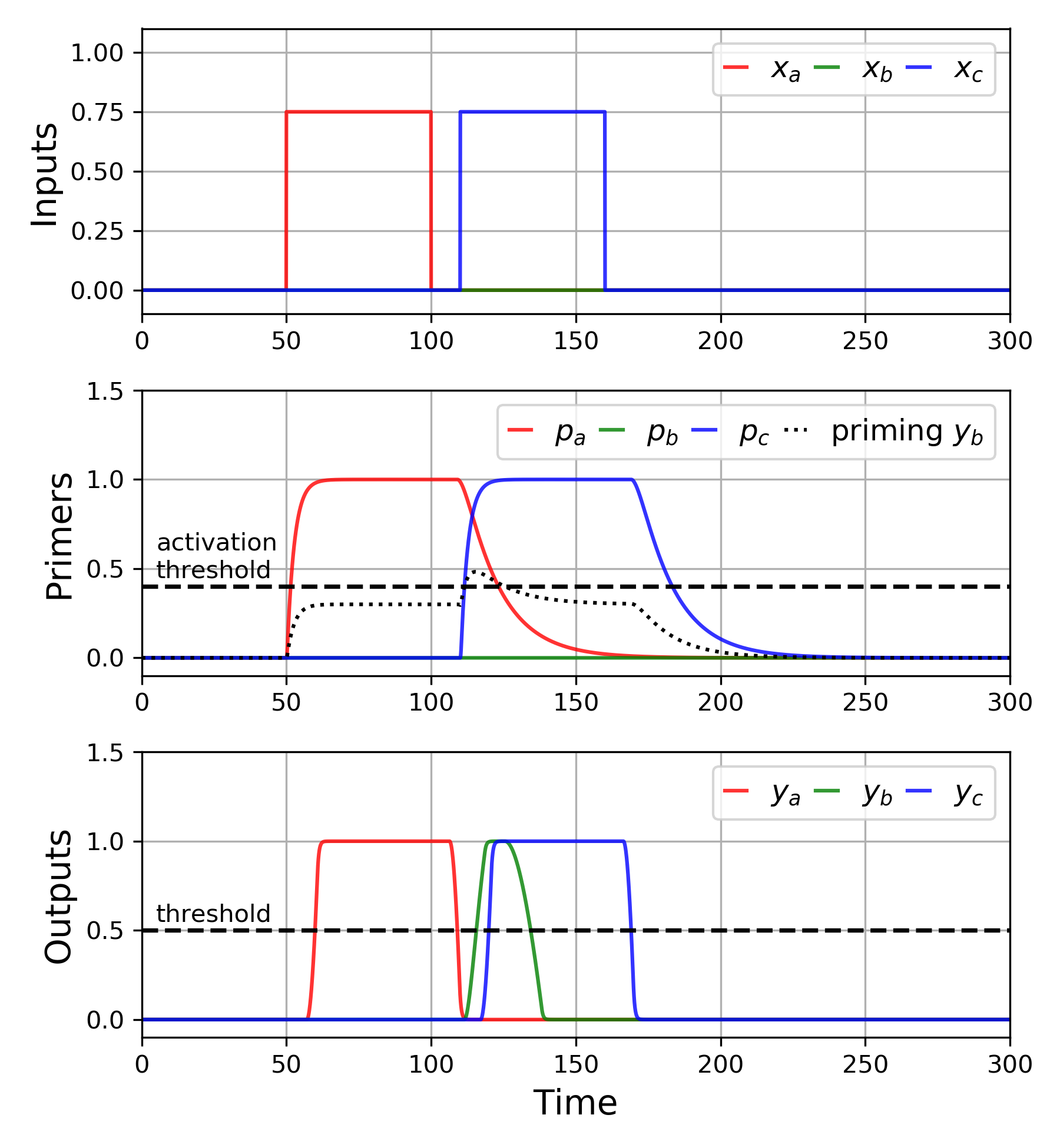

Supplement: S1 Data — (ZIP) [file pcbi.1009344.s005.zip › S1_Data/phi/Fig4d.png]

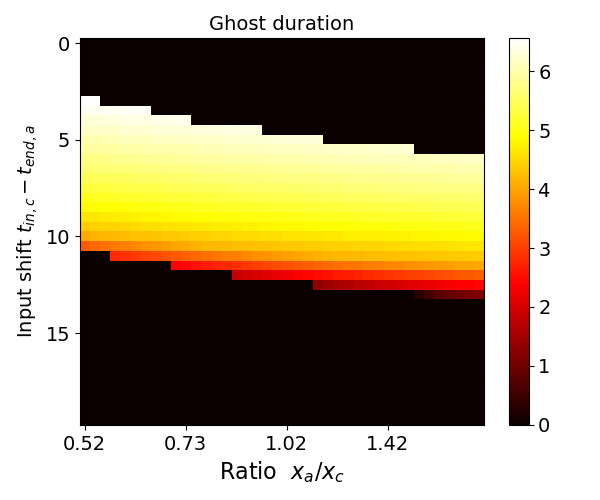

Supplement: S1 Data — (ZIP) [file pcbi.1009344.s005.zip › S1_Data/phi/extra_scan/phi_scan_amplitude_interval.png]

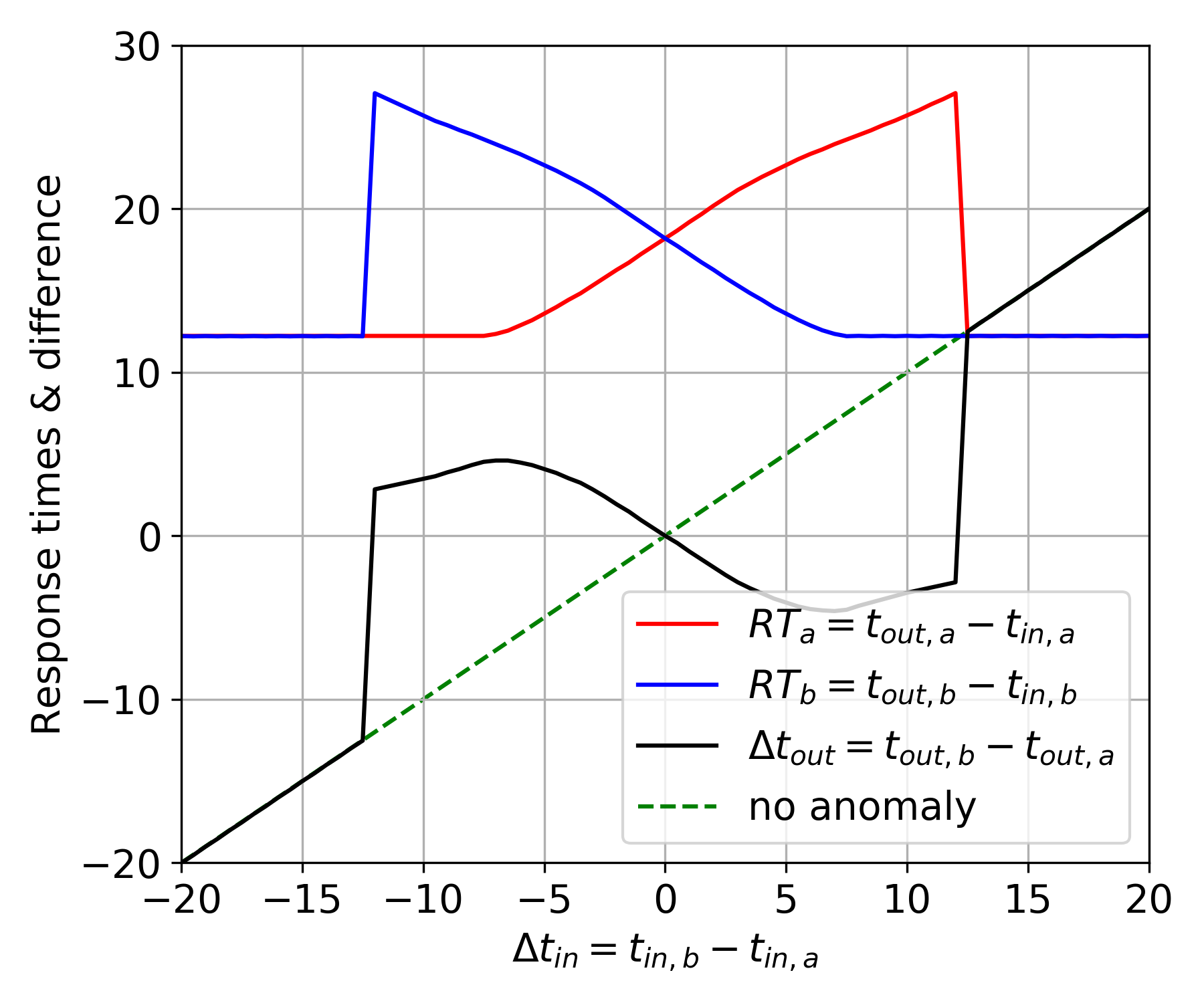

Supplement: S1 Data — (ZIP) [file pcbi.1009344.s005.zip › S1_Data/timereversal/Fig3b.png]

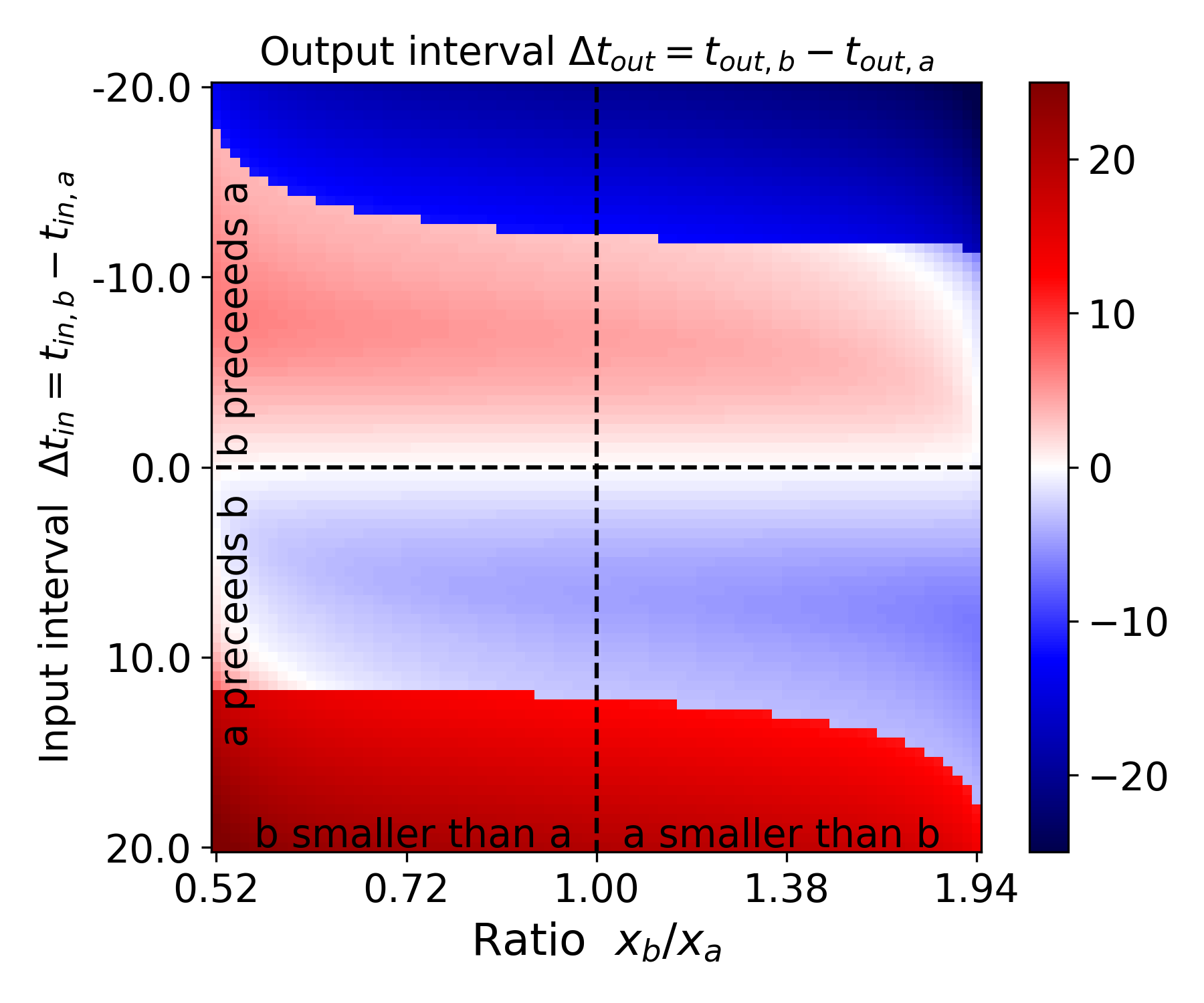

Supplement: S1 Data — (ZIP) [file pcbi.1009344.s005.zip › S1_Data/timereversal/Fig3c.png]

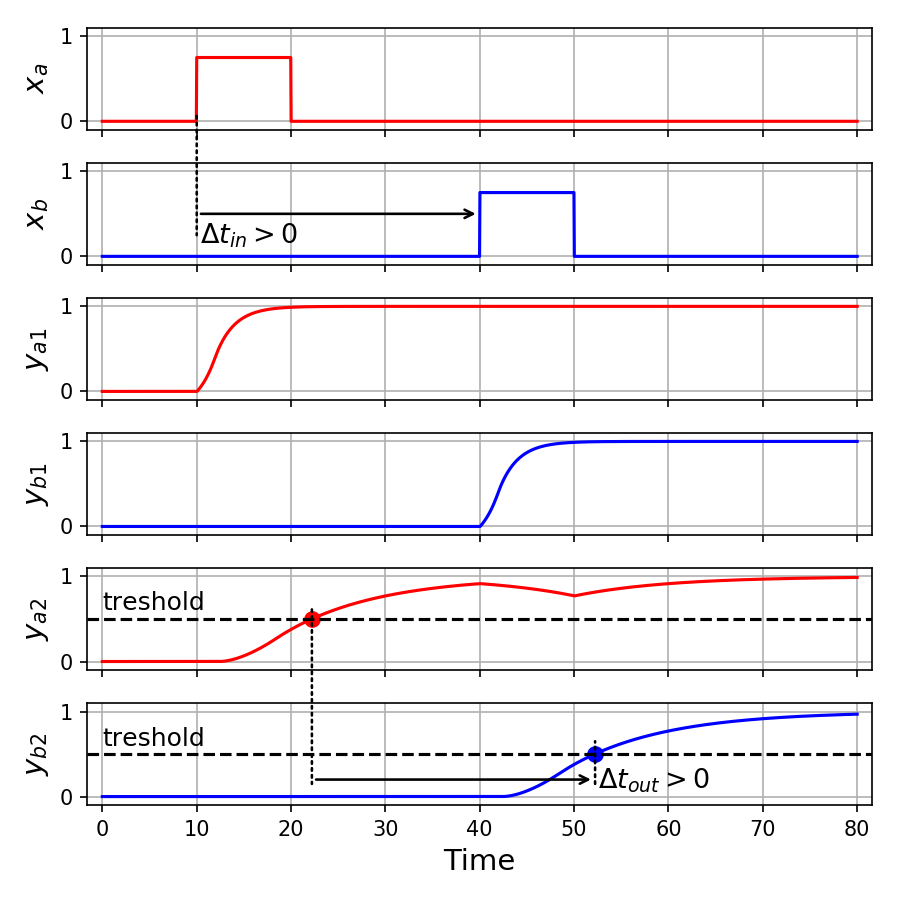

Supplement: S1 Data — (ZIP) [file pcbi.1009344.s005.zip › S1_Data/timereversal/Fig3d.png]

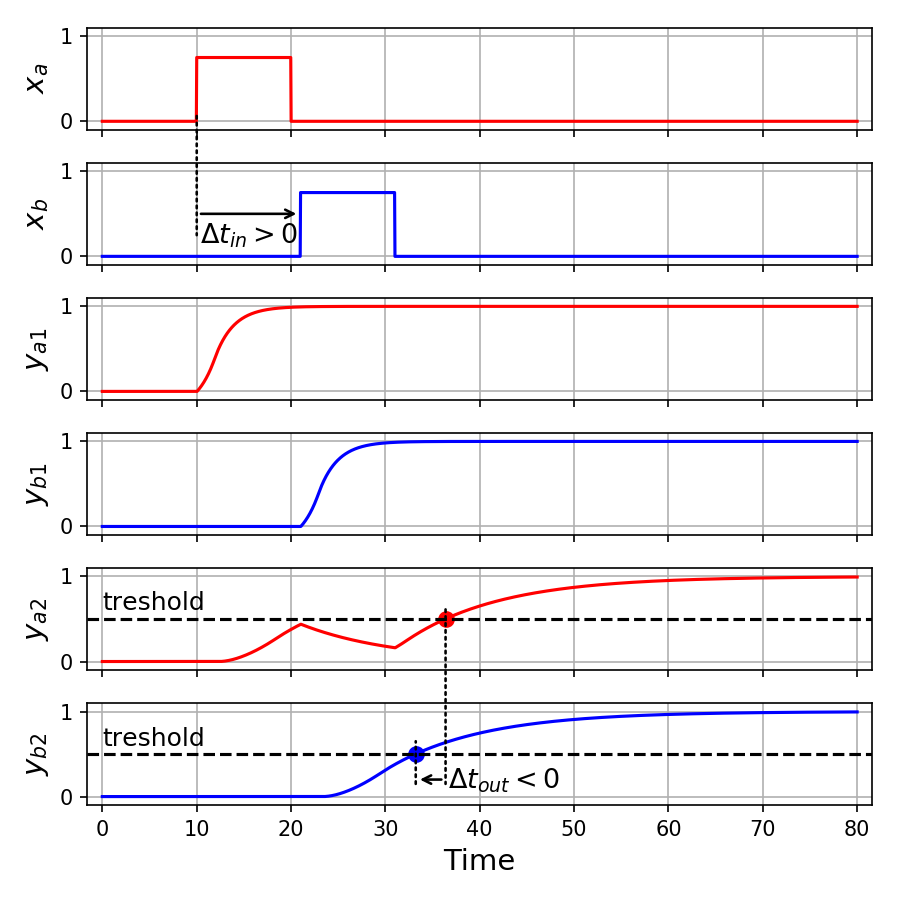

Supplement: S1 Data — (ZIP) [file pcbi.1009344.s005.zip › S1_Data/timereversal/Fig3e.png]
