# Supplementary material for: The color phi phenomenon: Not so special, after all?
Source: PLoS Comput Biol. 2021 Sep 3;17(9):e1009344. doi: 10.1371/journal.pcbi.1009344 (PMC8445478; doi:10.1371/journal.pcbi.1009344)
Supplement: Attachment — Submitted filename: Reply_AC_LK.pdf [file pcbi.1009344.s007.pdf]

Dear Editor,

We should like to begin by thanking you and the Reviewers for the careful handling and thorough examination of our manuscript “The color phi phenomenon: not so special, after all?” We find ourselves encouraged by the positive feedback of the Reviewers, who appear to judge that our work is both timely and interesting.

At the same time, the Reviewers made it clear that several shortcomings need to be addressed in our manuscript. We insufficiently stressed the main point of our work, namely that relatively simple bottom-up dynamical mechanisms operating fully outside consciousness *can* be an alternative account of a phenomenon that has previously been taken to constitute a demonstration of the postdictive nature of perception. The ad-hoc way in which we presented the time-reversal and phi-networks made the Reviewers wonder how they were constructed, and this therefore requires more explanation. Furthermore, the intriguing results provided by our echo state network experiment calls for more analysis as to their origins.

Below, we take the opportunity to address the Reviewers' concerns point-by-point. Our replies are italicized. As requested, we have prepared a revised version of the manuscript in which changes are indicated in color, as well as a clean updated version. These changes are also included in this letter. Finally, our simulation code has been added to the supplementary material.

We are convinced that the revisions detailed below have drastically improved the quality and readability of our work, and very much hope that you and the Reviewers will now be favorably disposed towards publication.

Kind regards,

Prof. Dr. Axel Cleeremans
Dr. Lars Keuninckx

Reviewer #1:

Review for article: The color phi phenomenon: not so special, after all?

The key idea in the article is that illusory percepts that are informed by later stimuli can arise from bottom-up network interactions, contrary to the common hypothesis that such perceptual illusions evidence "retrospective construction" in conscious perception. This intriguing hypothesis, if true, would offer an alternate explanation to how conscious perception arises.

Perceptual illusions such as the color phi phenomenon (CPP) have been taken as evidence that conscious perception is retrospective, or has retrospective elements, because such illusions involve percepts that are influenced by stimuli that are given later in time. For instance, in the color phi phenomenon one perceives an illusory percept between two differently-colored stimuli that rapidly flash onscreen (e.g. an illusory purple dot between a red and blue dot). Because the illusory percept's content is in-between the two real stimuli, and it is perceived before the second real stimuli, it exemplifies a percept that is informed by information later in time, potentially implying that conscious perception is retrospective.

The authors took another perspective; perhaps, such reversals are driven by bottom-up network interactions, such as local recurrence and long-reaching feedforward connections, rather than retrospective construction. They simulated simple neural network models to see whether the key aspects of CPP (order reversing of perceived stimuli, ghost stimuli, and illusory mixed-color stimuli) can be explained by dynamic network interactions.

After a brief example that demonstrates backwards masking in a simple network model, the authors demonstrate aspects of CPP from three different networks. First, they constructed a network that, through feedforward lateral inhibition, would reverse the order of stimuli in downstream activations if the input stimuli were spaced just right. Thus, this network demonstrates a time-reversal of stimuli, one of the key aspects of CPP. Next, the authors constructed a network that, through lateral feedforward excitation that would "prime" nearby outputs, could give rise to a "ghost" response between two input stimuli. Thus, this network demonstrates a phi phenomenon, also a key aspect of CPP. Finally, the authors constructed an echo state network (ESN), wherein a randomly connected network is used to encode different input signals, and then information about the input is decoded from the random network's activity using a trained decoder. With the right timing, delivering a "left red" and then a "right blue" signal would lead the network to a state where "red", "blue", and "middle" decoders were activated in between

the two stimuli, constituting a mixed-color ghost response. Thus, this network exhibits a phenomenon that resembles CPP.

The main strengths of this paper are that it demonstrates an intriguing alternate explanation to a well-accepted hypothesis about perception alongside proof-of-concept results, gives detailed explanations of network results and what they mean, and thoroughly explains the thought process that led them to their hypothesis. The findings detailed in this article will likely change the way we think about perceptual illusions and their implications for conscious perception. One of the paper's weaknesses are that the first two models for perceptual are fairly limited, and for the third ESN model only a small fraction of the randomly generated networks exhibited CPP, and the authors do not analyze what differs between the networks that exhibit CPP and those that don't. Another weakness is that the text has many ideas densely packed together into long paragraphs, especially in the introduction and discussion, making it difficult to follow at certain points.

We would like to thank the Reviewer for the encouraging comments.
Below we show how we have addressed his concerns step-by-step. First, we would like to stress the point that not only a mixed color perception (red+blue stimuli leading to purple perception) can happen, but a complete change of perception to the final color in a time-wise anomalous manner, which is a similar but, in our opinion, even stronger effect than the one we captured in our ESN model. In fact, showing a mix of colours (both red and blue outputs alongside the 'middle' output active) likely would have led to a significantly increased fraction of the ESN networks that showed the color phi phenomenon (CPP).

To clarify this in the revised manuscript, we made this change to the introduction:

"...the perception is that of the first dot moving to the position of the second dot and changing to the color of the second dot somewhere along the perceived path. The perception of a mix of the colors of the dots is also possible."

The time reversal and the phi models are indeed limited and very specialized in their performance. This was by design: we wanted to show how the CPP could be decomposed into two separate effects, each of them represented in small and explainable dynamical networks. It was our hope that the structure of these networks would lead to insights on how to combine them so as to capture the complete CPP. However, this proved more difficult than anticipated, which is why we turned to the echo state network (ESN) approach instead.

We added/changed:

"The models presented in the previous sections illustrate both aspects of the CPP as discussed. Unfortunately, there seems to be no straightforward way to combine their structure into a single CPP model. Therefore, we turned to neural network paradigms to find such a model."

Please note that, next to showing how the CPP could be driven by bottom-up nonlinear dynamical processing, we aimed at illustrating how nonlinear dynamics in general is a key ingredient in constructing models for effects in which time plays a central role.

Minor points:

1. In the abstract, the authors state "Our results suggest that similar illusions are likely to be obtainable for any system, artificial or biological in nature, with minimal neuron-like properties, and are thus merely artifacts of the inherent dynamical and nonlinear behavior of such systems" which is too strong of a statement given the limitations of their first two models and the rarity of ESNs that demonstrate CPP (1.2% of tested networks, lines 431-432). The findings are proof-of-concept for their hypothesis but do not provide evidence that that this is how perception actually functions (this would require some comparisons to real data). Please change the strength of this claim to be more in line with the final sentence of the article.

   We agree with the Reviewer that this statement is worded too strongly and have reworded as follows:

   "Our results suggest that the color phi phenomenon and similar illusions might simply be an artifact of the inherent dynamical and nonlinear processing present in networks with minimal neuron-like properties."

2. At the end of the section "ESN Experimental Results", lines 428-429, the authors say "Since the network consists of N = 200 dynamical 428 neurons, we cannot explain in detail how this behavior arises." Given point (1) above, it would greatly strengthen the article to know how the networks that do exhibit CPP differ from those that don't. For instance, perhaps the networks that exhibit CPP had more inhibitory connections or longer decay timescales by chance. Other ideas for analysis can be seen in Kim & Sejnowski (2020) (citation below), where they similarly analyzed a network close to an ESN. If the authors did run some comparisons and got null results, it would still be useful to know what factors they analyzed.

Indeed, we could only detect the CPP in a small fraction (adjusted to ~1.87% with extended experiments, see below) of the ESNs and had initially not attempted to analyze what exactly the differences are between the networks that do and those that do not show the effect. We reasoned that having shown the presence of the CPP in even a low number of small dynamical networks (a mere 200 neurons) was cause enough to bring into doubt its special status regarding human perception and consciousness. We stress that the low number of networks that did show the CPP-like effect is precisely caused by the fact that they were not trained explicitly to display it. Therefore, one could argue that for all of these networks the CPP was an artifact of dynamical processing.

We have added the following analysis about the possible internal differences between the ESNs that do and those that don't show the CPP effect. Unfortunately, it is a negative result: for the four chosen characteristics we checked (others than in the reference), no significant differences could be found.

*"**Analysis:***

Since the model is essentially a system of N=200 coupled nonlinear difference equations, we cannot explain in detail how this behavior arises. It is obvious, however, that simple dynamical interactions give rise to the existence of the CPP here.

Nevertheless, it is important to ask if there are any discernible differences between ESNs that do and those that do not show the CPP effect. In Fig. 8 we present four network characteristics that we specifically checked to identify such differences. The plots are based on N=100000 randomly assembled ESNs in total. In general, only a low fraction of the ESNs show the CPP. In our experiments this was 1.87%. Fig. 8(a) shows the distribution of α-parameters of the neurons. In Fig. 8(b) we plot the magnitude of the eigenvalues of the reservoir matrix W_{res}. Both of these metrics govern the timescales at which the dynamics evolve. There is no clear difference between networks with and without the CPP. In Fig. 8(c) we plot the ratio of excitatory (positive) to inhibitory (negative) synapses. Finally, Fig. 8(d) shows the number of positive long distance feedforward connections (LDFF) in the networks. Here, a connection from neuron i to neuron j is counted as LDFF, if i positively influences j via both a direct and an indirect path. This test was suggested by our phi model, where priming was caused by LDFF connections. Again, no differences between networks that show the CPP and those that do not come forward.

These negative results suggest -but of course do not prove- that it truly is the detailed internal structure that matters, and the ESNs are otherwise macroscopically indistinguishable. We suspect only a small portion of the neurons in the ESNs assists in generating the CPP."

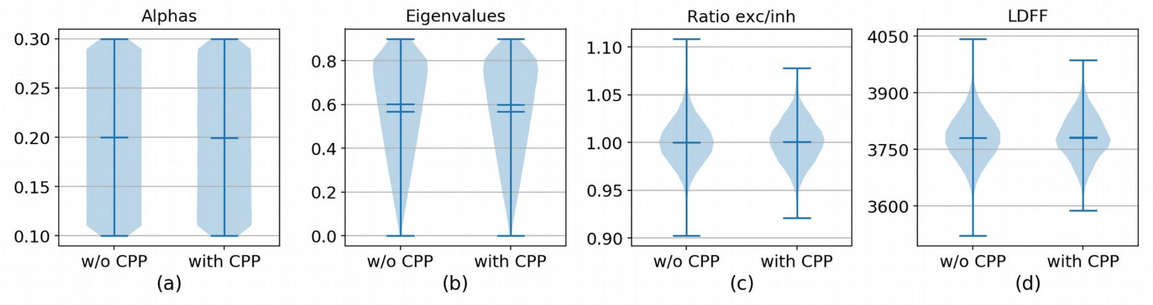

Figure 8: Some characteristics of the ESNs that do (N_{with}=1867) and do not ($N_{without}$=98133) show the CPP: (a) distribution of neuron α-timescales, (b) magnitude of the reservoir matrix eigenvalues, (c) ratio of excitatory to inhibitory synapses, (d) number of positive long distance feedforward connections.

a. If the authors elect to not run such analyses, it would be good to address limitations (points 1 and 2) in the discussion.

We elected to add a (non-exhaustive) analysis as described above and are grateful to the Reviewer for the useful reference. Regarding points 1 and 2, we augmented the discussion as follows:

"We stress again the crucial point that during the training phase of the ESN we explicitly excluded input conditions that could invoke a CPP: the readout layer was constructed from the network's responses to input stimuli that did not contain direct left-to-right or right-to-left jumps of opposite color. We found 1.87% of N=100000 networks showed the CPP, with the network parameters we chose. Kim and Senjowski [2021] showed that for a spiking recurrent neural network performing a working memory task, network characteristics that strongly influence the emerging timescales, and which are thus good predictors of performance, can be found. We similarly analyzed several characteristics of our ESNs, but failed to find markers that predicted the presence or absence of the CPP."

We further note that it is not a priori clear which neurons of an ESN are involved in the CPP effect: by the very nature of the technique of echo state networks, a good portion of the neurons (or in fact even a majority) might only deliver dynamical "clutter" which does not offer a noticeable contribution to the output signals at all. The importance of a particular neuron is not, in general,

ascertainable from the value of its associated readout layer weight, since a neuron with a low (absolute) readout weight value might still indirectly have a strong influence, via the neurons it is connected too.

3. It is unclear whether the section "Example: backward temporal masking" (line 154) constitutes original work or if it is an example from the literature. If it is original work, adding a sentence to clarify would be helpful. Alternatively, the authors could relegate this section to a supplementary section with a pointer from the text, because it serves mostly as an introduction to their methods and is not related to their question of interest. If it is from the literature, reference(s) should be provided.

   This section is original work. Our goal was to give the reader who is not familiar with nonlinear dynamics a feel for the subject, thereby reaching a larger audience. The introductory example demonstrates what is possible with even a single dynamical neuron, the key ingredient of course being time. Therefore we would rather not move this material to the supplementary section. To the best of our knowledge, this exact simple (and limited) model has not been discussed before in the context of sensory masking. On the other hand, we are sure that we are not the first to have demonstrated this idea in one form or in another. Our references include Mathis and Mozer [24], who posed the connection between stable states and conscious perception, using a model described in the language of difference equations. Commonly, stable states of a dynamical system (be it in the form of difference or differential equations) are interpreted as local minima of a scalar potential function. Locally these can be approximated by a parabolic function. This is what is shown in Fig. 2(b), where in this case no parabolic approximation is needed for the two minima, precisely because the example system Eq. (3) was chosen precisely to have a parabolic potential function. To make this more clear, we added the following in the Section "Example: backward temporal masking":

   "As discussed in the introduction, stable attractor states in the brain, at least temporary ones, have been hypothesized as the basis for conscious percepts [14, 24]. We emphasize that although the example given in this section is original work that to the best of our knowledge has not been shown earlier, it is hardly original in spirit and only serves as an introductory example."

4. The authors should break very long paragraphs up and provide more structure in order to improve readability (especially Introduction paragraph 1, Discussion paragraphs 1 and 2).

   We added several paragraph breaks in the introduction and discussion of the revised manuscript to make the text more readable.

References:

Kim R, Sejnowski TJ. Strong inhibitory signaling underlies stable temporal dynamics and working memory in spiking neural networks. Nature Neuroscience. 2021 Jan;24(1):129–39.

Reviewer #2:

Reviewer #2: The authors present an echo state model, which can capture the color phi phenomenon. The results are interpreted within the broader context of the philosophical questions about the time course of consciousness. The research questions is timely and interesting. The presentation, the specific research question, and the model in action were not always clear to me.

We thank Reviewer 2 for the detailed reading of our manuscript and the many challenging pointers on where we could improve. Below we reply to his concerns and show how we adapted the manuscript to address them.

Majors:

It is not easy to get what the model exactly does because the presentation is sometimes quite wooly and at other, important instances very short. For example, why presenting a model that does not work?

It is not directly clear to us which of the three models that we present the Reviewer is referring too. All three work and demonstrate -within their stated limitations- the described aspects of the CPP. However, on careful rereading, we noticed a confusing choice of words caused by an unfortunate literal translation. We had written "out of order" (as in "not working") where we meant "chronologically reversed". This has been fixed in the revised manuscript:

"In the next section, we show a small network of toy model neurons that illustrates the first part of the CPP, namely how perceptual states and their respective causal stimuli can appear to be chronologically reversed under certain circumstances."

Why having this lengthy detailed description of integrate and fire neurons? Why not explaining the echo state network right away and more clearly.

First, we must point out that the neuron model we used is the non-spiking leaky integrator model, not to be confused with the leaky integrate & fire (LIF).
The leaky integrator neuron is the natural dynamical extension of a simple perceptron: next to nonlinearity, another key ingredient we needed was time dependent behavior.
The reason we gave this lengthy description is because we wanted to give readers less familiar with nonlinear dynamics a brief and visual (hence the animations in the supplementary material) introduction. Understandably, for the reader who is more acquainted with this subject this introduction is redundant. We have emphasized this by adding the following sentence to this section:

"The reader who is familiar with nonlinear dynamics can skip the remainder of this section."

If I got it correctly, the model is more or less a low pass filter, which translates the input into a perceptual space producing some neural activity in the middle between, say the right blue and left red input, which mimics the phi phenomenon because there is activity at a location where nothing is produced. OK this is interesting.

We would be hesitant to use the term low pass filter for the ESN model itself. The input stimuli (three positional, each with two possible colors, so six in total) are decoded by the ESN model into position and a color at that position, so five output lines in total. In itself this is a trivial task, as long as the timings are respected that the network was presented with during training. The CPP-like response appears when direct transitions between left/red and right/blue are applied, at appropriately short intervals. This shows that the effect is driven by transient dynamics. To make this all a bit more clear we added an animation to the supporting material:

"The supporting information contains a short clip [S4_Animation] produced directly from the timeseries of Fig. 7(a), showing how the ESN 'perceives' the CPP. The perception of the final transition from left red to right blue briefly shows a blue dot in the middle."

Referring to the neuron model itself, the Reviewer is right that the leaky integrator model is a (nonlinear) low pass filter. However, a plethora of response types can be obtained from circuits assembled from this neuron. For example, a high pass behavior is formed by subtraction (via an inhibitory synapse): if x is an input signal and f(x) is a low passed version of x, then x-f(x) is a high pass of x. Fixed states and thus memory are found by above-unity feedback. The typical spiking of excitable systems can be had by cross-coupling two such neurons. We see the leaky integrator neuron as a convenient dynamical building block.

However, what are the implications? It seems that the authors want to attack certain notions about the time course of consciousness by Dennett and us by showing that a simple model can do the job. It remains however unclear to me what exactly they want to attack. Or maybe they do not want to criticize things? At other instances it reads like that the authors want to naturalize philosophical thoughts. Please clarify.
A simple motion detector can also detect apparent motion and even the phi phenomenon when equipped with a color detector. Thus, even more simple mechanisms may exist. What do we learn from the authors model then?

Indeed, we have not made our point sufficiently clear.
Regarding the above remark on the phi phenomenon, we are uncertain how to interpret it, since the phi phenomenon does not involve color. Therefore, we take it the Reviewer meant the color phi phenomenon and will reply with that in mind.

In the literature we only encountered conceptual explanations the CPP.
In contrast, we provide a quantitative dynamical connectionist model, which can be contrasted against experiments. Furthermore, we show that the temporal order reversal present in the CPP can be shown to exist in a particularly small network of only four neurons. This network, which is an alternate expression of a 4th-order nonlinear dynamical system, could easily exist within the brain at a functional level.

What we learn from this is that the bottom-up mechanisms we have shown could -and we emphasize could- be at least in part responsible for such phenomena as the CPP. Thus, stimuli that come in at a later time can not only block earlier stimuli, as in backward masking, they can also coexist in the perceptual space. Our models show that this scenario can come to play in a purely bottom-up manner.

We agree that a CPP-like effect may be shown in even smaller networks, having less than N=200 neurons. We have not been able -nor was it our goal- to isolate the active parts in the ESN that are responsible for the observed effect and it may turn out to be surprisingly small. As stated elsewhere in this reply letter, it is in the nature of the random

construction of ESNs that many neurons only provide dynamical clutter, and do not feed into the readout layer with a significant weight.

The implications of our work are that the CPP itself cannot longer be seen as proof of the post-dictive nature of perception.

It seems to me also that the network does not perceive motion, it just creates a signal in the middle of the trajectory, isn't it?

Indeed. Both for the phi phenomenon model and the CPP we approximated the visual system as a very coarse one-dimensional three-pixel perceptual space. For the phi model this kept things to an explainable level: fast acting feedforward priming connections where responsible for the "ghost" perception. For the ESN-based color phi model, the inputs were doubled to represent red and blue. We thought about a model having a more natural higher resolution visual input and output scene, as a follow up to this work.

There seems to be also a misunderstanding about our labels. These are not Dennett's time stamps. Our model claims that motion and other temporal aspects of a stimulus are rendered conscious at one short moment as a vector of feature labels, such as (Shape=line, duration=100ms, motion=l-r, color=red). Hence, the motion is not perceived during the 100ms of the presentation, not even for 100ms, just as a number, namely, the output of a duration detector. In this respect, it seems that the model is different from our model since it outputs a continuous stream.

This seems to be a difference indeed.
The notion of feature vectors containing time labels, as the Reviewer explains, is not necessarily at odds with our way of modeling. It is just so that our model does not incorporate such a notion at this time. We already remarked in the discussion of our manuscript that congruently with his model, our model shows continuous integration (and thus processing) of stimuli that are initially not reportable. The neurons in our model output a continuous real-valued stream. In the order-reversal model for example, we arbitrarily set the threshold of perception to halfway the output, at 0.5. It is straightforward to create a digital on/off signal based on exceeding this threshold, while constant integration of a signal related to the onset of the stimulus would yield a value proportional to time. This integrated value would then act as time label in the feature vector described by the Reviewer. The threshold acts as a gatekeeper of the time label (and the other features) into conscious perception. As we pointed out, this timing label is limited in the sense that it cannot represent an absolute or wall clock time. Its value must reflect a notion of time relative to the larger perceptual context or mental state, in other words related to the temporal positions of percepts in working memory. To make these

points clear, we adapted the discussion as follows:

"In line with Herzog et al. [2016], our simple model shows continuous feature integration and a by definition discrete, threshold-like rendering of features into conscious percepts. Additionally, timing labels may be constructed as neuronal activity, the value of which is related to the time since the onset of a (not yet reportable) stimulus. A thresholded signal derived from the stimulus as a whole acts as a gatekeeper. Naturally, a neural timing signal would have to be relative to the larger perceptual context or mental state, rather than present any objective wall clock time. In summary, the experience of time is just like any other percept: a construction. Our four-neuron time reversal model illustrates how little machinery is needed to falsify this experience. Thus we conclude that our models, if needed, could incorporate time label features as proposed by Herzog et al. [2016]."

I have the feeling the authors want to defend a extensionalism (Dainton, Stanford Encycl.) and is a bit in the spirit of other models for example by Piper. Here, there could by a real controversy. These things are spelled out in Herzog et al. (2020).

We are not proponents of extensionalism as discussed in the Dainton, referenced below. Our models operate on the basis of a continuous and (assumed) unconscious integration, followed by a discrete rendering into conscious percepts, when and if the signals answer to certain requirements (here the simple crossing of a threshold). In our models, we did not consider the transition from one perception to the next without any percept extant in the mean time, as in the discrete extentional case. If the perception of time is dictated by feature labels that are rendered conscious, loosely speaking, as any other feature, would then the discrete extentional model not necessarily lead to a compression of time for the agent, where absence of a percept would imply absence of time perceived?
The idea that we are advocating for an extentional account might have been caused by the low fidelity of our models in terms of number of inputs and output positions. The latter was purely out of modeling convenience and with the goal of ending up with explainable models, in so far this was possible.

We clarified in the discussion as follows:

"Following the breakdown of possible types of temporal consciousness in Herzog et al. [2020], we consider our models to come closest to the discrete retentional class, albeit with percepts of finite non-zero duration which is at least in part governed by the stimuli. In our ESN model, not all feature perceptions are updated simultaneously and this is where the CPP-like effect originates from: the "color" feature is updated (meaning; crosses a perceptual threshold) asynchronously from the "position" feature, see Fig.

7(b). Thus features can be updated all at once, without noticeable timing differences, but this is not a given, as it generally depends on the characteristics of the stimulus and probably also on the larger perceptual context. What our ESN model indicates is that the CPP is the result of dispersion between features that stimulus-wise belong to each other, but are processed by different pathways. Under the right conditions, subtle timing differences caused by the different velocities with which the features traverse the neural substrate cause a noticeable binding error."

I think also Dennett would agree with the model. His time stamps are for representation of time, a very different questions than the authors focus on.
In any case, I think the authors should make clearer what they are aiming for.
Our model is mainly inspired by post-dictive effects as they occur in feature fusion, feature fusion with TMS, the sequential metacontrast paradigm (SQM), and other where a later presented stimulus integrates with a previously presented one. Hence, the question is to what extent the model can address such phenomena, for example, a simple feature fusion percept where a red disk followed by a green disk produces are yellow disk.

In our model of the stimulus-respose order reversal, both percepts (outputs) represent a stimulus and both are present, so indeed there is no perceptual fusion.

In our ESN-based CPP model, we aimed at detecting the final color of the stimulus (blue) before the corresponding stimulus was perceived at the final location. These were the conditions the model was tested on, notably without being exposed to CPP-inducing input conditions during training. Had we probed for the condition of the "red" output to be active simultaneously with "blue", then this could have been construed as perceiving a mix of colors (likely with a higher yield of networks that show the effect). We are fairly confident that a model for transcranial magnetic stimulation (TMS) induced sensory fusion of colors could be constructed in a similar way, by disrupting the continuous integration of the stimuli as they are integrated.
We clarified as follows in the introduction (see also reply to the Reviewer #1 above) :

"...the perception is that of the first dot moving to the position of the second dot and changing to the color of the second dot somewhere along the perceived path. The perception of a mix of the colors of the dots is also possible."

To reiterate, our aim was to provide a quantitative account of how the CPP might be driven by bottom-up processes. Both the order reversal and phi phenomenon networks rely on local recurrences and long-distance feedforward connections. Although we could not identify the exact mechanism responsible for the CPP in the ESN model, it is clear that given the relatively small size of this network and the fact that it was assembled

using the same leaky integrator neuron building blocks, similar simple interactions are at play. In the discussion we added:

"Local recurrences evidently may induce long lasting states without the need for large buffers and feedforward priming connections, as discussed in the section on the phi phenomenon model, can be interpreted as a form of prediction."

Minors:

"The core aspect of what needs to be explained in the case of the CPP is the subjectively experienced reversal of the order in which stimuli occur." Why reversal? There is no reversal in app motion. The first disk is perceived before the second one.

Indeed, this was sloppily formulated. We rephrased as:

"The core aspect of what needs to be explained in the case of the CPP is the chronological anomaly it presents us with: how can the color of the second stimulus be experienced before its position is?"

"We conclude that the CPP, while an intriguing visual illusion in its own right, might be nothing more than a consequence of the inherent dynamics that characterize information processing in the brain." The CPP is not an inherent dynamic. It may be explained by one. What is the argument here? That Dennett has claimed that it needs sophisticated processing?

To emphasize, we did not write in that the CPP is an inherent dynamic, as that would make no sense indeed. We wrote that the CPP is perhaps <u>a consequence</u> of such dynamical behavior (line 502 of the original manuscript). On the contrary, we claim that sophisticated modeling is not required, and that is exactly what we have shown with our models. To the best of our abilities, we could not find a connectionist, time-based model of the CPP presented in the earlier literature.

Our main argument is that the CPP might be less informative about human consciousness than once claimed, because it can be construed as a side effect of finite-speed (hence dynamical) bottom-up processes, fully outside the scope of awareness.

What do we learn from the subplot "neuronal state" in fig 6?

The neuronal states of Fig. 6 merely illustrate how the entire network responds to the input with what is in the reservoir computing field loosely referred to as "rich temporal dynamics". Plotting all two hundred neurons might have been a bit too much, so we limited the plot to the first ten, which gets the point across much better:

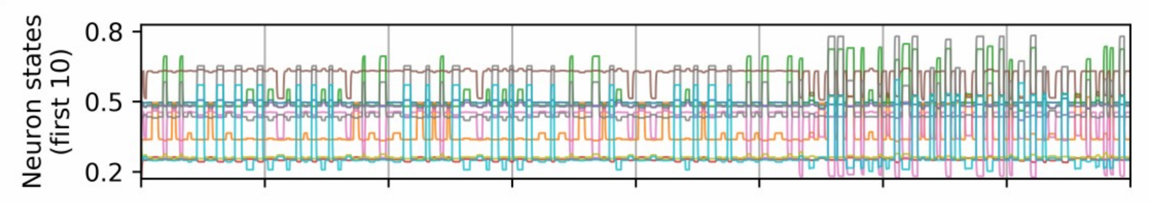

"After training, i.e. calculating the readout layer $W_{out,}$ we can show the resulting actual output in response to the training input (bottom graph of Fig. 6). The output is assembled from the complex network dynamics (first ten neurons shown in second graph of Fig. 6)."

English is not always clear: For example: "When undriven by a stimulus ~x(t), the state \ falls" downward towards a local minimum of and scalar function V (~y) : R^K -> R, much like a golf ball rolling downward to the lowest point of a valley it happens to find itself in." Undriven = no stimulus presented. States don't fall. I think state is the wrong word here because at each time point of "falling" is a state. I think a reader who understands the Nabla Operator knows what an attractor state is and thus needs no metaphors. For those who do not know, I am not sure whether the metaphor helps.

We tried to visualize what happens, the word "falling" referring to the analog situation of a mass seeking a minimum in a gravitational field. Undriven could also mean after the stimulus has returned to zero, as in the backward temporal masking example: in Fig. 2(c) and (d), after the stimuli have returned to zero, the state still evolves towards an equilibrium as a consequence of said stimulus:

"When undriven…, the state evolves downward towards a minimum of a scalar function..."

Indeed, an attractor state will be clear to anyone who has some knowledge of nonlinear dynamics. However it was our intention to also reach readers who may not be all that familiar with this subject and specifically its usefulness in modeling cognitive phenomena. Those readers may find this introduction useful, even only to trigger their interest. We reference Strogatz's ground-breaking introduction to the subject. To make it clear that the informed reader can skip the section, we added:

“The reader who is familiar with nonlinear dynamics, can skip the remainder of this section.”

Mathematically: why is a global minimum reached and not a local one?

We did not mention a global minimum, so we are unclear as to what is referred here. Relating to the golf ball analog “the lowest point of a valley” in general is a local minimum but could indeed be the global minimum (we envisioned the typical golf course with several valleys). The exact classification of the minimum depends on the potential function involved. We removed the word “local” to avoid any confusion.

"Since it is not straightforward to manually construct a neuronal circuit that shows the complete CPP, we turn to neural network paradigms to find one." Rather void sentence.

We agree. A better motivation is required here. We replaced this sentence with:

“The models presented in the previous sections illustrate both aspects of the CPP as discussed. Unfortunately, there seems to be no straightforward way to combine their structure into a single CPP model. Therefore, we turn to neural network paradigms to find such a model.”

Lines 166 ff could be removed. This should be clear to readers of Plos Comp.

We intended to trigger the interest of the reader less acquainted with nonlinear dynamics and its many applications, modeling cognitive phenomena a somewhat lesser highlighted one. As explained above, the informed reader can skip the section, thus we added:

“The reader who is familiar with nonlinear dynamics, can skip the remainder of this section.”

Figures are low resolution. Please present them in the text together with the captions.

Indeed, something went wrong while assembling the pdf of the initial submission and we apologize for this oversight. Our original figures were of high resolution. Please note that we left out the figures in the text because we explicitly followed the instructions in the header of the Latex template of PloS. We apologize if this was inconvenient to the Reviewers.

"merely artifacts of the inherent dynamical and nonlinear behavior of such systems." Why artifacts? Again, it seems that authors want to say "look we do not need complicated machinery" but is unclear who they think claimed otherwise.

Note that the ESN was trained to decode the inputs, red/blue at positions left/middle/right, without having seen transitions that could lead to a CPP (Fig. 6, top graph): no direct transitions such as left/red to right/blue were presented in the training set. The calculation of the output after training, based on the responses to the training stimuli (Fig. 6 bottom graph), showed that, qualitatively, the network performs as intended (compare to the third graph "ideal output"), however it is not perfect: there are artifacts for the latter part of the training set that consists of confusing inputs for which the outputs should ideally be zero. These are artifacts of the dynamical processing that happens in the network.

As it turns out for some of these networks, when driven by left/red to right/blue stimuli with decreasing interval, an effect having all the features of the CPP is seen at the outputs. Since -and this is key- the networks were not trained for this, this effect is an artifact of the dynamical processing.

To the best of our abilities, we could not find any other quantitative dynamical and connectionist CPP model in the current literature. We take this as a sign that the community is convinced that such modeling is challenging and complicated machinery would be needed (the alternative explanation, that there is no interest in this topic, we did not take serious). We ourselves were surprised by the relatively straightforward way in which these results could be obtained, especially for the very small stimulus-response order reversal network. Our model offers a baseline from which comparisons to experiments can be derived.

"In Ref. [7]," spell out please.

This was changed to:

"...as argued by Cleeremans and French [17]..."

Mathis and Mozer, no year is given.

This was changed to point to the right reference:

"Mathis and Mozer [24]"

Signed
Michael Herzog

Dainton, B. (2018) Temporal consciousness. In The Stanford Encyclopedia of Philosophy Winter 2018.

Michael H. Herzog, Leila Drissi-Daoudi, and Adrien Doerig (2020).
All in Good Time: Long-Lasting Postdictive Effects Reveal Discrete Perception. TICS.

Piper, M.S. (2019) Neurodynamics of time consciousness: an extensionalist explanation of apparent motion and the specious present via reentrant oscillatory multiplexing. Conscious. Cogn.

Reviewer #3:
The authors address what is on the face of it an intriguing mystery, the so-called color-phi illusion which has been argued to evidence postdictive processing or even discrete perception by some. Rather than offering yet another conceptual analysis, the authors take a much more sensible approach to the problem, and set out to show that this phenomenon could result from generic facets of the dynamics of artificial neural networks.

The authors do well in presenting the rational of their (and similar) modeling approach, stressing the logic in first demonstrating the general nature of an effect in abstract modeling. As a modeling device, the authors choose to utilize leaky integrator neurons, which are exhibit several of the properties one would want from neural building blocks not the least of which is a temporal aspect to their response. They proceed to present three simple models in increasing order of complexity, a model for temporal reversal, a model for the phi phenomenon, and a model incorporating both.

The authors modelling efforts and analysis are a timely addition to the recent discussion triggered by Herzog et al's discrete model of perception. As such they expose a possible weakness in the original argument, namely that time reversal has to be construed as postdiction. However, I think the paper would benefit from some additional analyses as listed below before it is published:

We thank the Reviewer for his thorough examination of our manuscript and for the encouraging words. Below we show how we address his comments.

Major comments:

I really like the later parts of the discussion. I think they highlight some major issues with the notion of imposing discreetness on neural dynamics, and the conceptual link between discreteness and consciousness. I recommend that this somewhat agnostic view be represented also in the earlier discussions.

In light of the comments received from prof. Herzog, who also is Reviewer #2 of this work, we clarified in the conclusion that we are not opposed to a model in which at the (very least) some aspects of perception work in a discrete manner: after all, we take an arbitrary value of 0.5 (halfway the range) at the output of our models as the threshold of perception.

Indeed, our models do not lead us to a strong proof of how perception works (no model or even set of models on a single cognitive phenomenon could be asked to do that) and we believe that, if eventually found, a collection instead of single mechanism will be involved.

We see mathematical modeling as nothing but the logical extension of an argument, leading to a notion of how something could be, as elaborated on in "From chicken squawking to cognition", Ref. [17] of the original manuscript.

As we have now clarified in the discussion, our way of modeling, which in itself is not very new, is not incompatible with a form of time label features which are treated as percepts themselves. After all, there is no argument that a notion of time that is associated with the more tangible aspects of a physical stimulus, is itself a perception. These time labels, as we argument in our original manuscript, cannot for practical reasons reference some absolute wall clock time. They must somehow map to relative times in the entire perceptual context they belong too. Here it is simply the case that, as far as the CPP goes in our models, these time labels are not needed. We repeat the clarification from above:

"In line with Herzog et al. [2016], our simple model shows continuous feature integration and a by definition discrete, threshold-like rendering of features into conscious percepts. Additionally, timing labels may be constructed as neuronal activity, the value of which is related to the time since the onset of a (not yet reportable) stimulus. A thresholded signal derived from the stimulus as a whole acts as a gatekeeper. Naturally, a neural timing signal would have to be relative to the larger perceptual context or mental state, rather than present any objective wall clock time. In summary, the experience of time is just like any other percept: a construction. Our four-neuron time reversal model illustrates how little machinery is needed to falsify this experience.

Thus we conclude that our models can be made compatible with the ideas proposed by Herzog et al. [2016]."

As suggested, we will also mention this earlier in the introduction:

"Also, we remain agnostic as to the existence of feature vectors that include time labels as proposed in Herzog et al. [2016]; our models do not require them as far as the CPP goes, but could be made compatible with that idea."

We feel that the waters are much muddier regarding the transition between percepts. To the discussion we added:

"In our models, we (naively?) interpreted the end of a percept as coinciding with crossing the same threshold as its onset but in the opposite direction. The animation [S4_Animation] in the supplementary material of how the ESN 'perceives' the CPP, based on the timeseries of Fig. 7, is constructed in this way. However, as many experiments have shown, there clearly is an asymmetry in the sensitivity of when something emerges vs. when it disappears from our perception, even in very simple visual scenarios. Such hysteresis effects have been hypothesized by Poltoratski and Tong [2014] to disambiguate visual perception during the processing of scene transitions. In other words: the hysteretic thresholds are themselves a function of the scene in its entirety. Since a continuous signal -the precursor of a conscious percept- cannot pass two unequal values instantaneously, the existence of hysteresis naturally implies percepts of non-zero duration. Hysteresis effects could be explored further using the modeling approach shown in this article."

Poltoratski S, Tong F. Hysteresis in the dynamic perception of scenes and objects. J Exp Psychol Gen. 2014 Oct;143(5):1875-92.

We feel that the relatively simple mechanistic modeling we present, drives our main point home: perception as far as the CPP is concerned may be caused entirely by bottom-up dynamical processing before consciousness is involved. Thus the CPP cannot be held as strong evidence for anything relating to consciousness.

Order reversal model: The authors say that the parameters had to be handpicked to achieve reversal – but why couldn't they use simulation, and parametrization to produce a phase map to demonstrate the robustness of the effect?

Here we might have given of the wrong impression that the order reversal model was the result of a lengthy trial and error period, when in fact it was found mostly by reasoning

directly on what elements should be present: the circuit had to be symmetrical such that it responded in a symmetrical fashion and one branch had to influence the other branch in an inhibitory way as to slow it down, so negative cross-coupling was needed. Local positive feedback allowed for locking of a past stimulus. Most important, the cross-coupled connections had to jump forward in the chains, such that they could intercept the information of the opposing chain, as it flowed from input to output: the key idea was to exchange time for space. There were not that many architectural choices left after taking into account these boundary conditions. For the weight and bias parameters we picked simple numbers. The neuron tau-values where chosen such that the stimulus would be detected quickly in the input neurons of both branches, but would only slowly be integrated on the output neurons. In the revised manuscript we clarify this as follows:

"The key ingredient is the set of cross coupled inhibitory connections which jump forward in the chains, such that information that arrives at a later time can intercept partially processed information at the opposing chain as it flows from input to output. Loosely speaking, we make an exchange of time for space."

Regarding a phase map to assess the robustness of the reversal effect, we added to Fig. 3 a new plot, in which we map the output interval as a function of the input interval and the ratio of the stimuli (another possibility would have been the duration of the input pulses, which was kept equal here). Note this new plot does not replace Fig. 3(b), since that plot also shows the influence of the stimulus intervals on the response times, a slowing down of the responses when two stimuli are temporally close to each other. We added:

"Fig. 3(c) shows how the model behaves if we vary both the amplitudes of the input stimuli and the interval between them. Here, the sum of the amplitudes is kept at 1.5 and their ratios are varied. In Fig. 3 (c), the lighter colored central part marks output intervals that are temporally reversed relative to the input interval. There is a tendency, within certain limits, that the more stronger a later arriving stimulus is, the easier it can overtake an earlier one. Thus the model demonstrates how both temporal and amplitude-related characteristics of complex stimuli influence their temporal perception in a non trivial way."

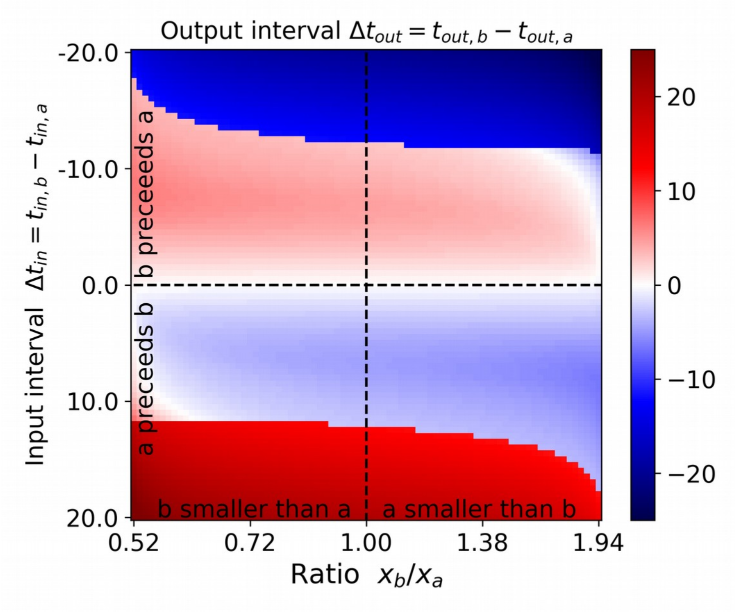

“Fig3. (...) (c) Scan over stimuli intervals and amplitude ratios, illustrating how temporal and amplitude-related stimuli characteristics influence their temporal perception.”

And in the discussion, we changed the text as follows:

“We see several directions in which we could expand this work. First, it is clear that the interactions that take place in the networks we proposed are influenced by the amplitude, duration and interval characteristics of the stimuli, something we did not fully explore, except briefly for the introductory masking example and in Fig. 3(c). In the latter it was shown that the relative strength of the stimuli influences the temporal perception of their intervals. Results from such explorations could be compared to empirical data with the goal of finding similar scaling laws.”

The authors acknowledge the limitations of their phi phenomenon model, but again do not directly assess the robustness of even this limited result (the movie is a step in the right direction).

The Reviewer correctly points out that we did not further explore the phi phenomenon model. The phi model was only intended to showcase how fast feedforward priming could generate ghost perceptions. For the time-reversal model, as detailed above, a further exploration gave the insight that temporal perception can be influenced by both stimulus amplitude and interval in a nontrivial way.

For the case of the phi phenomenon model, next to the interval, the amplitudes and input widths of the input stimuli will affect the fast acting feedforward priming paths and thus also the possible appearance of a ghost. However, we feel this is hardly surprising. Our code in the supplementary material contains a script, phi_scan_amplitude_interval.py, that prepares a map of ghost durations as a function of stimulus strength ratio and interval between them:

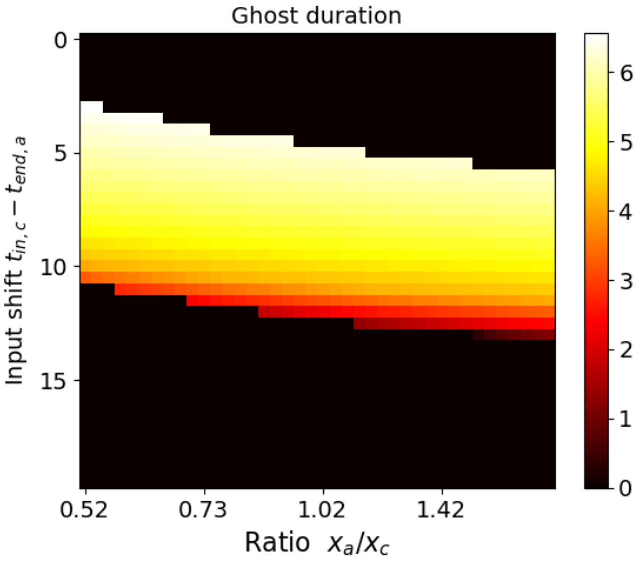

Here the interval is defined as the time between the end of the 'left' and the beginning of the 'right' stimulus. In the interest of saving space, we left out this result from the revised manuscript. Note these results are also heavily influenced by the neuron weight parameters and should only be interpreted in a qualitative way. We added to the phi phenomenon section:

"Similar to the case of the order reversal model, as shown in Fig. 3 (c), the stimulus characteristics influence whether or not a phi phenomenon happens. Our code in the supplementary material contains a script that plots the duration of the ghosts as a function of stimulus interval and amplitude ratio. "

Again in the CPP model we are not offered insight as to robustness – e.g. what is the effect of the sparseness parameter? The spectral radius? Etc. (i.e. do we still get CPP in 1.2% of random networks?)

Indeed, we did not examine the ESN's parameters further.
Please note Reviewer #1 asked if we could pinpoint the differences between those ESNs

that do and those that didn't show the CPP. As outlined above in this reply, we have added such an analysis (for the current network parameters). Unfortunately, that approach did not offer any insight, at least not for those network characteristics we tested. We suspect that only a small portion of the ESN is active in generating the CPP, such that any macroscopic characteristic that is derived from the network's parameters is unable to catch it. Reverse-engineering the active part of such ESNs is not a trivial task.

The Reviewer might wonder how we got to the ESN parameters such as sparsity and spectral radius in the first place. The values we chose are quite typical for ESNs. We did not try to optimize them. The following reference was added:

"Network hyperparameters were chosen as suggested in Ref. [Lukoševičius 2012]."

Lukoševičius M. In: Montavon G, Orr GB, Müller KR, editors. A Practical Guide to Applying Echo State Networks. Berlin, Heidelberg: Springer Berlin Heidelberg; 2012. p. 659–686. Available from: https://doi.org/10.1007/978-3-642-35289-8_36.

As the Reviewer suggests, it would be interesting to have an idea of how robust the results are regarding the network parameters. We agree, even if it were only to find better values that can increase the yield and thus speed up future explorations.
To the section on "Analysis" of the ESN, we added:

"In Fig. 9, we show a brief exploration of how robust the results are with respect to the choices of network parameters. Here, we scan over reservoir and input sparsity, spectral radius and input scaling, the latter being the σ-value with which the input matrix W_{in} is constructed. The non-varied parameters are as described in the previous section. Each point is the fraction p out of n=200 ENSs that showed the CPP. The error bars are estimated at the 95% confidence interval using Wald's method.

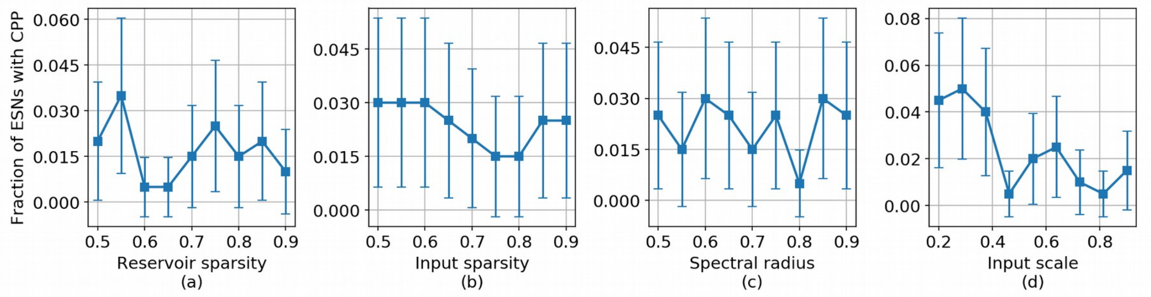

Fig. 9: ***Fraction of ESNs that show the CPP as a function of several network parameters.*** *(a) input sparsity (b) reservoir sparsity (c) spectral radius (d) input scaling. Each point represents n=200 tested networks. The error bars are estimated at the 95% confidence interval. Non-varied parameters kept as described in the text.*

It must be remarked that invariably such parameters are highly interdependent, such that for the purpose of optimization they must be probed together. We did not explore this any further as this is was outside of the scope of this article."

From Fig. 9, there is some variance in the yield of CPP-showing ESNs, but no spectacular changes are seen. Similar sensitivity can be expected when varying the stimulus parameters.

We stress again that the fact that the yield of networks that show the CPP was quite low, does not refute our claim: even if just one such (relatively small) ESNs could be demonstrated, our conclusion, that the CPP could be nothing more than an artifact of dynamical processing unrelated to consciousness as such, would stand.

Minor comments

"A four toy model neuron model" a four neuron toy model?

We thank the Reviewer for catching this, and have changed to:
"A four-neuron model".

I'm not sure eq. 5 helps illustrating the point – it just complicates a very straight forward example.

Eq. 5 functions as a generalization of the example. It also links the models in the remainder of the manuscript with others who took a similar dynamical approach such as Mathis and Mozer[24] and many others. In that sense we feel it is useful, even if somewhat abstract.

Why does the output layer have to be conceived as a cartesian theater? Can't it be taken to be as behavioral output, thus implying that sensory motor mapping is the substrate of representation?

Indeed this could be the case as in a real CPP experiment, the subjects would have to report on their experience. Thus there is always an element of motor activation or at least planning. We added:

"Alternatively, the outputs of the readout layer could be seen as a behavioral response."

Why is the ESN constrained not to respond to simultaneous stimuli?

The input scenes of a real CPP experiment are in and of themselves not complicated (single dot and position) and it is not clear how a participant in an experiment is supposed to react to a confusing scene (multiple dots shown simultaneously or two dots of different color at the same position). Not presenting such conditions to the network during training would have been unrealistic, so they must be mapped to some output vector. We felt it is natural to choose zero, equivalent to "no response", for this.

As an aside - our recent paper offers some analysis and points to existing evidence from VSDI to the proposed mechanism for phi illusions (In the interest of saving time: a critique of discrete perception)

The paper mentioned above is indeed relevant for our article. It was added to the references. In the discussion we wrote:

"In our ESN model, not all feature perceptions are updated simultaneously and this is where the CPP-like effect originates from: the ``color'' feature is updated (meaning; crosses a perceptual threshold) asynchronously from the``position'' feature, see Fig. 7(b). Thus features can be updated all at once, without noticeable timing differences, but this is not a given, as it generally depends on the characteristics of the stimulus and probably also on the larger perceptual context. What our ESN model indicates is that the CPP is the result of dispersion between features that stimulus-wise belong to each other, but are processed by different pathways. Under the right conditions, subtle timing differences caused by the different velocities with which the features traverse the neural substrate cause a noticeable binding error.

Fekete et al. [2018] have argued at length against a perception that is tied down in a ``discrete straightjacket'', on the basis of both empirical physiological data and

philosophical arguments. One strong argument against a fully discrete awareness is the asynchronicity of inter area communications of the brain.”

Have the authors made all data and (if applicable) computational code underlying the findings in their manuscript fully available?

The PLOS Data policy requires authors to make all data and code underlying the findings described in their manuscript fully available without restriction, with rare exception (please refer to the Data Availability Statement in the manuscript PDF file).
The data and code should be provided as part of the manuscript or its supporting information, or deposited to a public repository. For example, in addition to summary statistics, the data points behind means, medians and variance measures should be available. If there are restrictions on publicly sharing data or code —e.g. participant privacy or use of data from a third party—those must be specified.
Reviewer #2: Yes
Reviewer #3: None

Have all data underlying the figures and results presented in the manuscript been provided?
Large-scale datasets should be made available via a public repository as described in the PLOS Computational Biology data availability policy, and numerical data that underlies graphs or summary statistics should be provided in spreadsheet form as supporting information.
Reviewer #1: No: The article was a simulation study, so no data was collected. They say they will include the python code in a zip file in the supplementary material, but that is not done with this manuscript.

In the revised submission, we have added our simulation code in the supplementary material. The Python scripts directly generate the figures of the manuscript.
In the interest of reproducability, where random initialization is used (for example in the generation of the ESN reservoir matrices), the random number generators were pre-seeded with a fixed value, such that the scripts always generate the same values in the same order.
