# Supplementary material for: The color phi phenomenon: Not so special, after all?
Source: PLoS Comput Biol. 2021 Sep 3;17(9):e1009344. doi: 10.1371/journal.pcbi.1009344 (PMC8445478; doi:10.1371/journal.pcbi.1009344)
Supplement: Attachment — Submitted filename: Reply_AC_LK_3.pdf [file pcbi.1009344.s009.pdf]

Dear Editor,

We would like to thank you, the other members of the editorial team and the Referees for accepting our manuscript. Below, we address the final points that Reviewer #2 brought up and that have prompted us to carry out some further minor changes and clarifications in the text.

We would like to emphasize that we are grateful for the many challenging comments that the referees have provided us with. They have forced us to think long and hard about the meaning of our computational results (which we humbly admit are up for debate).

Once again, our replies are italicized. Changes to the previous submission are represented in blue in this letter. Further, we happily accept that the peer review history be made publicly available at the time of publication.

Kind regards,

Prof. Dr. Axel Cleeremans
Dr. Lars Keuninckx

Reviewer #2:
The authors have clarified and improved many things. Still, I am not really happy with the presentation. The main problem is the imbalance of context, which makes it hard to get what is important. Basic, well known stuff is explained in great detail and other things, such as the Nabla operator (I guess most readers are not familiar with), are just mentioned en passant (the fix "readers familiar..." does not really work). I will not urge the authors to change it, if they do not want to. It is their paper.

We again thank the Reviewer for his interesting comments.

The importance of Eq. (5), the one containing the Nabla operator, is that it sets the context for the kind of models that we apply: ordinary differential or difference equations (ODEs) (admittedly, we do not prove the existence of the scalar potential function V for our models). This links our work to the existing body of research that has taken a similar route, such as Mathis and Mozer [24]. This is not a trivial matter. In the previous review, Reviewer #3 mentioned the technique of voltage-sensitive dye imaging (VSDI). Looking at VSDI recordings, one instinctively thinks about the spreading of activation that is made visible with this technique in terms of a diffusion process, typically described by a more complicated and inherently infinite-dimensional partial differential equation (PDE). Thus one could argue that at first glance PDEs might be more suited to model (a part of) the extremely high-dimensional system that is the brain. However, we wanted to drive home the point that the color-phi phenomenon can be a captured in small systems of low dimensionality and is thus unrelated to consciousness as such. Eq. (5) sets this context.

We regret that the Reviewer indicated that in some instances we are overly explaining simple things and at others are moving too fast. The reason for this is, in our opinion, clear: our paper lives on the uneasy boundary between nonlinear dynamical modeling and cognition. We feel that no matter how we formulate our case, it will remain very challenging to close this gap to everyone's approval. To emphasize the important points, we further edited the Author Summary, and changed the format to a bullet points style:

- *Well known visual illusions are used to investigate how objective physical stimuli translate to subjective conscious experiences and are therefore believed to offer clues at how human consciousness works.*
- *One such illusion, the color phi phenomenon is marked by an anomalous time reversal of what is shown to test subjects and what they report to have seen.*
- *The color phi phenomenon has been a prime example to illustrate that conscious experience, instead of being merely a recording of the facts as they happen, involves both pre- and post-constructive processes resulting in a coherent narrative.*
- *We provide an alternative interpretation by showing that all aspects of this phenomenon can be adequately modeled in small neural networks. These networks are composed of simple leaky integrator neurons, emphasizing the importance of taking into account dynamical behavior.*
- *We illustrate how anomalous temporally-reversed perception of stimuli can appear based exclusively on bottom-up dynamics.*
- *Since the color phi phenomenon emerges as a feature of dynamical processing in our model, we conclude that it may explain less about human consciousness than previously thought, and that it could be nothing more than a feature of dynamical processing in the brain.*

The second thing it is still with the interpretation what the implications are. For example, in the abstract it is written that the “color phi phenomenon is…an artifact of inherent dynamics". This does not make to much sense to me. Why an artifact and not a feature? It seems the authors what to attack certain positions, but which ones? The term postdiction is indeed often used for mechanism rather than a term for psychophysical effects. What do the authors have in mind?

Indeed, the difficulty lies with the interpretation of the results that our models bring. First we again remark that our models offer a baseline mathematical description against which experimental results can be contrasted. To the best of our knowledge, such models have not been described elsewhere regarding the CPP.

Since the previous review, we have given it some more thought and indeed agree that naming the CPP an artifact might be uncareful: after all, we do not know which, if any, advantage the CPP

provides to the agent. To clarify: in our echo state network (ESN) model, the CPP emerges as an artifact or side effect of the dynamical processing, whereas for cognition, the CPP might be a (useful?) feature of the pre-cognitive sensory processing.

To this end we, we made these changes in the manuscript:

"Abstract: We show how anomalous time reversal of stimuli and their associated responses can exist in very small connectionist models. These networks are built from dynamical toy model neurons which adhere to a minimal set of biologically plausible properties. The appearance of a "ghost" response, temporally and spatially located in between responses caused by actual stimuli, as in the phi phenomenon, is demonstrated in a similar small network, where it is caused by priming and long-distance feedforward paths. We then demonstrate that the color phi phenomenon can be present in an echo state network, a recurrent neural network, without explicitly training for the presence of the effect, such that it emerges as an artifact of the dynamical processing. Our results suggest that the color phi phenomenon might simply be a feature of the inherent dynamical and nonlinear sensory processing in the brain and in and of itself not related to consciousness."

As stated in the discussion section, the CPP might be called a binding error caused by the differing processing speeds of a real aspect of a sensory stimulus (the final blue color) and an implied constructed perception (the "middle" dot). These get linked into an objectively anomalous perception. Note that if this is indeed the case, then a whole host of new CPP-like effects might be discovered, maybe even across modalities, by carefully combining stimuli for which we know that processing speeds markedly differ.

Regarding the term postdiction, in the Reviewer's recent TICS article "All in Good Time: Long-Lasting Postdictive Effects Reveal Discrete Perception", postdictive effects are defined as:

"in postdictive effects, a late stimulus can affect perception of another stimulus presented earlier. In certain postdictive effects, earlier and later stimuli can even mutually affect each other. This is different from other temporal effects such as priming, in which earlier stimuli affect perception of later ones (but not the other way around)."

We would like to point out that with this definition, backwards temporal masking, as in the simple example in our article (Fig. 2) is a postdictive effect: the late stimulus (mask) affects the perception of the earlier one (the prime).

Now, as per above definition, this effect is different from priming, yet when the prime in Fig. 2 (b) of our article is strong enough, then this objectively earlier stimulus will affect the perception of the later one: the mask will not be perceived and the prime will be. In other words: the mask is what is getting masked by the prime (note here the mask is above-threshold), and their roles have effectively been reversed.

What it comes down to is this: at least in this example, what is the mask and what is the stimulus cannot be pinpointed at in a clear-cut unambiguous way, based solely on their objective temporal order, and is subject to the interpretation of the experimenter. Therefore, we feel that a definition of postdiction that is based on the temporal order of the stimuli is lacking something.

The difficulty in defining postdiction in a general context is that it needs to take into account elements of both objective stimuli and their subjective perception (or lack thereof), the latter being a construction of which the objective temporal properties are uncertain (except for those that stem from causality, of course).

The question thus remains as to what level a stimulus needs to be processed, that is formed into a (partial?) perception before it can be said that it partakes in or drives a postdictive effect. Can any feedback loop, even of a single spike, be deemed the neuronal substrate of a postdictive effect? If one chooses to answer this question in an affirmative manner, then all neuronal processing is postdictive, something we certainly do not advocate for.

We see postdictive effects as feedback effects that exist very late in the processing, critically being driven by neuronal states which will, at that point, inevitably give rise to reportable perceptions. We are aware that this is not a clean definition.

What we argue in our article is that as far as the CPP goes, no processing that is unique to consciousness is needed. The effect can be explained by simple neuronal dynamics alone, long before consciousness needs to be involved.

Reviewer #3:
The authors did a thorough job in addressing the reviewers comments, thus I gladly endorse it for publication.

We thank the Reviewer again for the encouraging comments and interesting pointers.

Additional changes:

-we mentioned the code in the Supporting Information:
"Folder 'code' Contains the Python scripts that directly generate the graphs of this article."

-in the Acknowledgments, we changed:
"AC is a Research Director with the Fonds de la Recherche Scientifique F.R.S.-FNRS (Belgium)."
