# Supplementary material for: The color phi phenomenon: Not so special, after all?
Source: PLoS Comput Biol. 2021 Sep 3;17(9):e1009344. doi: 10.1371/journal.pcbi.1009344 (PMC8445478; doi:10.1371/journal.pcbi.1009344)
Supplement: Attachment — Submitted filename: CPP_review.docx [file pcbi.1009344.s006.docx]

Review for article: The color phi phenomenon: not so special, after all?

The key idea in the article is that illusory percepts that are informed by later stimuli can arise from bottom-up network interactions, contrary to the common hypothesis that such perceptual illusions evidence “retrospective construction” in conscious perception. This intriguing hypothesis, if true, would offer an alternate explanation to how conscious perception arises.

Perceptual illusions such as the color phi phenomenon (CPP) have been taken as evidence that conscious perception is retrospective, or has retrospective elements, because such illusions involve percepts that are influenced by stimuli that are given later in time. For instance, in the color phi phenomenon one perceives an illusory percept between two differently-colored stimuli that rapidly flash onscreen (e.g. an illusory purple dot between a red and blue dot). Because the illusory percept’s content is in-between the two real stimuli, and it is perceived before the second real stimuli, it exemplifies a percept that is informed by information later in time, potentially implying that conscious perception is retrospective.

The authors took another perspective; perhaps, such reversals are driven by bottom-up network interactions, such as local recurrence and long-reaching feedforward connections, rather than retrospective construction. They simulated simple neural network models to see whether the key aspects of CPP (order reversing of perceived stimuli, ghost stimuli, and illusory mixed-color stimuli) can be explained by dynamic network interactions.

After a brief example that demonstrates backwards masking in a simple network model, the authors demonstrate aspects of CPP from three different networks. First, they constructed a network that, through feedforward lateral inhibition, would reverse the order of stimuli in downstream activations if the input stimuli were spaced just right. Thus, this network demonstrates a time-reversal of stimuli, one of the key aspects of CPP. Next, the authors constructed a network that, through lateral feedforward excitation that would “prime” nearby outputs, could give rise to a “ghost” response between two input stimuli. Thus, this network demonstrates a phi phenomenon, also a key aspect of CPP. Finally, the authors constructed an echo state network (ESN), wherein a randomly connected network is used to encode different input signals, and then information about the input is decoded from the random network’s activity using a trained decoder. With the right timing, delivering a “left red” and then a “right blue” signal would lead the network to a state where “red”, “blue”, and “middle” decoders were activated in between the two stimuli, constituting a mixed-color ghost response. Thus, this network exhibits a phenomenon that resembles CPP.

The main strengths of this paper are that it demonstrates an intriguing alternate explanation to a well-accepted hypothesis about perception alongside proof-of-concept results, gives detailed explanations of network results and what they mean, and thoroughly explains the thought process that led them to their hypothesis. The findings detailed in this article will likely change the way we think about perceptual illusions and their implications for conscious perception. One of the paper’s weaknesses are that the first two models for perceptual are fairly limited, and for the third ESN model only a small fraction of the randomly generated networks exhibited CPP, and the authors do not analyze what differs between the networks that exhibit CPP and those that don’t. Another weakness is that the text has many ideas densely packed together into long paragraphs, especially in the introduction and discussion, making it difficult to follow at certain points.

**Minor points:**

1. In the abstract, the authors state “Our results suggest that similar illusions are likely to be obtainable for any system, artificial or biological in nature, with minimal neuron-like properties, and are thus merely artifacts of the inherent dynamical and nonlinear behavior of such systems” which is too strong of a statement given the limitations of their first two models and the rarity of ESNs that demonstrate CPP (1.2% of tested networks, lines 431-432). The findings are proof-of-concept for their hypothesis but do not provide evidence that that this is how perception actually functions (this would require some comparisons to real data). Please change the strength of this claim to be more in line with the final sentence of the article.
2. At the end of the section “ESN Experimental Results”, lines 428-429, the authors say “Since the network consists of N = 200 dynamical 428 neurons, we cannot explain in detail how this behavior arises.” Given point (1) above, it would greatly strengthen the article to know how the networks that do exhibit CPP differ from those that don’t. For instance, perhaps the networks that exhibit CPP had more inhibitory connections or longer decay timescales by chance. Other ideas for analysis can be seen in Kim & Sejnowski (2020) (citation below), where they similarly analyzed a network close to an ESN. If the authors did run some comparisons and got null results, it would still be useful to know what factors they analyzed.
   a. If the authors elect to not run such analyses, it would be good to address limitations (points 1 and 2) in the discussion.
3. It is unclear whether the section “Example: backwards temporal masking” (line 154) constitutes original work or if it is an example from the literature. If it is original work, adding a sentence to clarify would be helpful. Alternatively, the authors could relegate this section to a supplementary section with a pointer from the text, because it serves mostly as an introduction to their methods and is not related to their question of interest. If it is from the literature, reference(s) should be provided.
4. The authors should break very long paragraphs up and provide more structure in order to improve readability (especially Introduction paragraph 1, Discussion paragraphs 1 and 2).

References:

Kim R, Sejnowski TJ. Strong inhibitory signaling underlies stable temporal dynamics and working memory in spiking neural networks. Nature Neuroscience. 2021 Jan;24(1):129–39.
